# Programming CAR T Cell Tumor Recognition: Tuned Antigen Sensing and Logic Gating

**DOI:** 10.1158/2159-8290.CD-23-0101

**Published:** 2023-03-24

**Authors:** Mohamad Hamieh, Jorge Mansilla-Soto, Isabelle Rivière, Michel Sadelain

**Affiliations:** 1 Center for Cell Engineering, Memorial Sloan Kettering Cancer Center, New York, New York.; 2 Immunology Program, Sloan Kettering Institute, New York, New York.; 3 Molecular Pharmacology Program, Sloan Kettering Institute, New York, New York.; 4 Cell Therapy and Cell Engineering Facility, Sloan Kettering Institute, New York, New York.

## Abstract

The success of chimeric antigen receptor (CAR) T cells targeting B-cell malignancies propelled the field of synthetic immunology and raised hopes to treat solid tumors in a similar fashion. Antigen escape and the paucity of tumor-restricted CAR targets are recognized challenges to fulfilling this prospect. Recent advances in CAR T cell engineering extend the toolbox of chimeric receptors available to calibrate antigen sensitivity and combine receptors to create adapted tumor-sensing T cells. Emerging engineering strategies to lower the threshold for effective antigen recognition, when needed, and enable composite antigen recognition hold great promise for overcoming tumor heterogeneity and curbing off-tumor toxicities.

**Significance:**

Improving the clinical efficacy of CAR T cell therapies will require engineering T cells that overcome heterogeneous and low-abundance target expression while minimizing reactivity to normal tissues. Recent advances in CAR design and logic gating are poised to extend the success of CAR T cell therapies beyond B-cell malignancies.

## INTRODUCTION

The advent of T cell engineering and CD19 chimeric antigen receptor (CAR) therapy has opened a new field of cancer immunotherapy (1, 2). Premised on genetic programming and synthetic receptors for antigen, T cells can be targeted to antigens other than HLA–peptide complexes and repurposed at will for a multitude of tasks. CARs typically engage cell-surface molecules through immunoglobulin-derived polypeptides, such as a single-chain variable fragment (scFv) and single-domain heavy chains (VHH), or other ligand–receptor interactions. Upon target engagement, CAR signaling not only activates but augments T cell functions through composite signaling modules (3, 4). CAR T cells targeting CD19, a cell-surface antigen found in most lymphomas, leukemias, and also normal B cells, represent the paradigm for this cellular therapy (1, 5). A series of remarkable clinical results obtained a decade ago in patients with refractory B-cell malignancies, including non-Hodgkin lymphoma (NHL), chronic lymphocytic leukemia (CLL), and acute lymphocytic leukemia (ALL), fostered worldwide interest in CAR T cell engineering and resulted in the regulatory approval of CD19 CAR therapies within a few years (6–12). Rapid progress has since been made in developing similarly conceived CAR T cells for the treatment of refractory multiple myeloma (MM) by targeting B-cell maturation antigen (BCMA; refs. 13–16). There are, as of this writing, six FDA-approved CAR products, four of which target CD19 and two BCMA (Table 1), and over 1,300 clinical trials utilizing a CAR molecule listed on the ClinicalTrials.gov website.

**Table 1.** FDA-approved CAR T cells

Target antigen	Name	Signaling design	Vector	Disease	Year
CD19	Tisagenlecleucel (Kymriah; Novartis)	4-1BBζ	Lentiviral	ALL	2017
				DLBCL	2018
	Axicabtagene ciloleucel (Yescarta; Kite/Gilead)	CD28ζ	γ-retroviral	DLBCL	2017
				FL	2021
	Brexucabtagene autoleucel (Tecartus; Kite/Gilead)	CD28ζ	γ-retroviral	MCL	2020
				ALL	2021
	Lisocabtagene maraleucel (Breyanzi; Juno/Bristol Myers Squibb)	4-1BBζ	Lentiviral	DLBCL	2021
BCMA	Idecabtagene vicleucel (Abecma; Celgene/Bristol Myers Squibb)	4-1BBζ	Lentiviral	MM	2021
	Ciltacabtagene autoleucel (Carvykti; Janssen)	4-1BBζ	Lentiviral	MM	2022

Abbreviations: DLBCL, diffuse large B-cell lymphoma; FL, follicular lymphoma; MCL, mantle cell lymphoma.

The prevailing CAR structures in use comprise an activation domain, typically the cytoplasmic domain of the CD3ζ chain, fused to the cytoplasmic domain of either CD28 (17) or 4-1BB (18), two costimulatory receptors that affect activation strength, effector and proliferative capabilities, apoptosis, and metabolism in the engineered T cells (3, 19). We hereafter refer to these two canonical structures as 28ζ and BBζ. In the context of CD19 CAR therapy, they provide overall comparable clinical outcomes (20). Most CAR T cells are generated by transducing the CAR cDNA into autologous T cells using γ-retroviral or lentiviral vectors (21).

Although this CAR design and manufacturing approach has yielded extraordinary results in some refractory hematologic malignancies, the direct application of the same CAR T cell recipe to solid tumors has not been as compelling (19, 22). Satisfyingly, however, a number of limitations to the effectiveness or applicability of CAR T cells have been identified, including antigen escape, which is to be expected in the face of tumor heterogeneity, insufficient T cell persistence, T cell functional decline, on-target/off-tumor toxicities, and the elevated cost of manufacturing autologous CAR T cells (23–26). These limitations provide a road map for research efforts aiming to improve future CAR therapies for both hematologic and solid cancers.

We focus here on how to enhance tumor recognition by CAR T cells and produce such cells. The targeting of a single antigen is fraught with limitations and pitfalls, exposing to primary resistance or relapse of tumor cells that either fully lack or express low levels of the target antigen. Strategies to enhance recognition of targets of low abundance and to enable T cells to safely and effectively engage more than one antigen are direly needed.

## ANTIGEN SENSITIVITY AND CAR DESIGNS

Considerable knowledge has accumulated over the past years on the 28ζ and BBζ foundational CAR designs, both of which achieve impressive activity when targeting CD19 (1, 20). These two canonical structures nonetheless differ in how they support T cell function, including the induction and maintenance of effector functions as well as T cell persistence and metabolism, resulting in differences in CAR T cell kinetics and toxicity profiles. These features have been amply analyzed in multiple reviews (3, 19).

**Figure 1.**
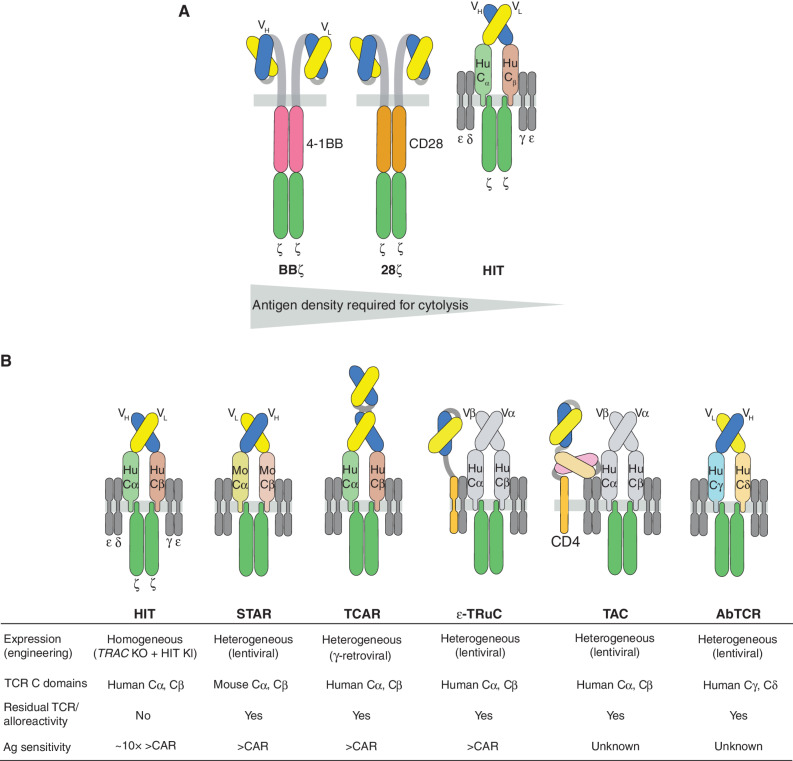
Structural design and sensitivity of CARs and CD3 complex–based receptors. **A,** CARs encompassing the 4-1BB (left) and CD28 (middle) costimulatory domains exhibit distinct antigen sensitivity *in vitro* and *in vivo*, with CD28-based CAR having superior antigen sensitivity (i.e., requires lesser antigen density for cytolysis). T cells expressing an HLA-independent TCR (HIT) receptor (right) that contains the same V_L_ and V_H_ domains require 10-fold lower antigen density than CD28ζ. **B,** CD3 complex–based receptors. HIT, synthetic TCR and antigen receptor (STAR), TCR-like CAR (TCAR), and antibody-TCR (AbTCR) receptors are based on fusing antibody variable domains to TCR constant regions (human Cα, Cβ for HIT and TCAR; mutated mouse Cα, Cβ for STAR, and human Cγ, Cδ for AbTCR). STAR, TCAR, and AbTCR are expressed using lentiviral or γ-retroviral, which results in heterogeneous expression. HIT T cells are engineered by targeting a V_H_-Cβ-P2A-V_L_-Cα (exon1) transgene into the TRAC locus, which leads to the disruption of the endogenous TCR and expression of the HIT receptor depending on the endogenous promoter and polyA signal. In ε-TRuC T cells, an scFv–CD3ε fusion is overexpressed using lentiviral vectors. This fusion is expected to compete with the endogenous CD3ε to get incorporated into the full TCR–CD3 complex. The TAC receptor is formed between the endogenous TCR–CD3 complex with a fusion composed of two scFvs (in tandem) and a truncated CD4 (lacking the MHC interacting domain), with one scFv specific to the antigen and the other specific to CD3ε, which leads to TCR–CD3 activation. Except for HIT T cells, all these T cells are expected to express residual TCR, which can lead to alloreactivity. Ag, antigen; KI, knockin; KO, knockout.

More recently, it has been recognized that 28ζ and BBζ CARs differ in their antigen sensitivity (Fig. 1A; refs. 27–30). In experimental models of ALL, Hamieh and colleagues observed leukemia relapses exhibiting reduced CD19 levels, which were more effectively treated with CD19.28ζ (19.28ζ) than CD19.BBζ (19.BBζ) CARs (27). In this model, the decreased CD19 levels measured at the time of relapse (<5,000 molecules per cell) were not due to the selection of CD19-null variants (31, 32) but were reversible and the consequence of CAR-mediated trogocytosis, a process whereby the target antigen is actively transferred from the tumor cell to the CAR T cell (27, 33, 34). When facing leukemia cells expressing 1,500 CD19 molecules per cell, 28ζ CARs showed superior *in vivo* leukemia control relative to BBζ CARs (27). When comparing the antigen sensitivity of 28ζ and BBζ CARs targeting either CD19 or GPC2, Majzner and colleagues and Heitzeneder and colleagues found that the 28ζ CAR better controlled *in vivo* tumor cells that expressed 2,000 CD19 or 6,000 GPC2 molecules per cell, respectively (28, 29). These findings are consistent with faster and larger changes in protein phosphorylation following antigen binding mediated by the 28ζ endodomain compared with BBζ (35). Although there may be exceptions depending on the epitope or the binder's affinity, these studies collectively establish that 28ζ CARs are better suited to target tumors with antigen levels <6,000 molecules per cell, whereas BBζ CARs are better suited for targeting tumor cells expressing high antigen levels and averting reactivity to normal cells only expressing few thousand target molecules per cell. Although 28ζ CARs have a lower target antigen density requirement, they too eventually struggle *in vivo* when target antigen density drops below 1,000 molecules per cell (36).

The sensitivity limits of canonical CARs raise the specter of tumor antigen escape and call for strategies to adapt CAR T cell design to specified ranges of target antigen density. Hereafter we discuss two promising remedies, one consisting in regulating the threshold antigen density for eliciting effective T cell activation and the other in generating T cells capable of productively engaging two or more target antigens.

### Setting the Threshold for Productive Antigen Recognition

CARs that fail to effectively control antigen-low tumors can be rendered more sensitive by modifying the CAR structural design or by parallel engineering to amplify their signaling. Thus, BBζ CAR sensitivity can be augmented by duplicating the CD3ζ chain segment to double the number of immunoreceptor tyrosine-based activation motifs (ITAM; ref. 28). Deleting ITAMs in BBζ and 28ζ CAR T cells decreases *in vitro* cytolysis of antigen-low tumors (28). Interestingly, however, point mutations in distal ITAMs in 28ζ do not diminish T cell function but to the contrary extend T cell persistence and delay terminal T cell differentiation in leukemia, pancreatic cancer, ovarian cancer, and melanoma models (37–39). The antigen threshold for eliciting cytolysis by BBζ CARs can be lowered to 5,000 molecules per cell by incorporating CD3ε (BB-εPRS-ζ) or the LCK binding site GRB2 (BBζ-GRB2; ref. 40). CAR components other than the signaling domain may also regulate the threshold of T cell activation. Thus, substitution of the CD8α hinge-transmembrane (H/TM) region of a BBζ CAR with the CD28-H/TM segment (17) further lowers the threshold for CAR reactivity, owing, at least in part, to the establishment of a more effective immunologic synapse (28, 29). Nonetheless, 28ζ CARs comprising CD28-H/TM still show superior control of antigen-low tumors relative to BBζ CARs reinforced by the CD28-H/TM domain (29).

CAR antigen sensitivity may also be modulated through the CAR's binding affinity (41). Cryogenic electron microscopy structural studies performed on the FMC63 and SJ25C1 CD19-specific scFvs have pinpointed contact residues with CD19 and guided the rational design of 28ζ CARs with increased or decreased sensitivity to CD19 (42).

CAR antigen sensitivity may also be increased without a structural modification by augmenting downstream activation signaling. Genome-wide CRISPR knockout identified RAS GTPase–activating protein (RASA2) as a checkpoint in T cells. Ablation of RASA2 expression in CAR T cells enhanced MAPK signaling and T cell cytolytic activity against a range of CD19-low tumors (43).

Much like the physiologic T cell receptor (TCR), CARs can display enhanced sensitivity by increasing their functional avidity for their target cell. This may be achieved through antigen-specific or antigen-agnostic pathways. The coexpression of a companion scFv in a CAR T cell can lower the antigen threshold for tumor lysis imparted by the CAR. Thus, expressing a cell-surface binder for CD38, with or without a signaling domain, lowers the threshold for cytolysis of BCMA or CD19 CARs in the face of low-abundance target antigen (44). Expression of ICAM-1 in T cells and their target has also been found to stabilize CAR synapse formation, much like for the TCR (45), enabling better lysis of some solid tumors (46).

Although tumor lysis *in vivo* is affected by additional tumor-intrinsic and microenvironmental factors, calibrated *in vitro* cytoxicity assays provide a useful metric for characterizing CAR T cell-intrinsic tumor recognition properties. Using tumor cells with graded CD19 levels, we further found that CAR T cells at an effector:target (E:T) ratio of 2 were more likely to lyse tumor cells with low target abundance than at a ratio of 1, suggesting the potential for cooperative killing when higher intratumoral E:T ratios are achieved (27).

### HIT Receptors and Other CD3 Complex–Based CARs

Virtually all CARs in present clinical development operate independently of the multimeric CD3 complex, which provides the signaling machinery to support TCR-mediated antigen recognition and signaling (47). In αβ-T cells, the antigen-binding αβ heterodimer associates with the signaling CD3 subunits δε, γε, and ζζ, which in aggregate provide 10 ITAMs per αβ heterodimer. *In vitro* T cell activation occurs upon the engagement of only a few cognate MHC–peptide complexes, underscoring the formidable signal transduction afforded by the TCR–CD3 complex (48). We investigated whether incorporating the V_H_ and V_L_ regions used in a sensitive 19.28ζ (27) into a TCR–CD3 complex increases antigen sensitivity relative to the V_H_–V_L_ matched CAR. We generated an HLA-independent TCR termed HIT receptor (Fig. 1A and B) by substituting chimeric V_L_–Cα and V_H_–Cβ chains for the endogenous TCR (36). Peripheral blood T cells expressing the HIT receptor displayed ∼10-fold greater antigen sensitivity than matched 28ζ CAR T cells and achieved control of leukemia expressing ∼200 CD19 molecules per cell in a mouse xenograft model. Using a comparable HLA-independent TCR comprising V_H_–Cα and V_L_–Cβ subunits called synthetic T cell receptor and antigen receptor (STAR; Fig. 1B), Liu and colleagues showed greater antigen sensitivity compared with 28ζ and BBζ T cells containing the same V_L_ and V_H_ elements (49), although CAR target densities were not quantified. Birtel and colleagues recently reported a TCR-like receptor containing V_H_–V_H_–Cα and V_L_–V_L_–Cβ subunits called TCAR (TCR-like CAR; Fig. 1B; ref. 50), which suggested increased sensitivity compared with a 28ζ and BBζ in terms of IFNγ secretion.

Even though chimeric receptors based on the TCR were proposed over 30 years ago (51), interest in CD3-based CARs is recent. In addition to the HIT and STAR receptors, TCAR, antibody-TCR (AbTCR; Fig. 1B), TCR fusion constructs (TRuC; Fig. 1B), and T cell antigen coupler (TAC; Fig. 1B)receptors also co-opt the CD3 complex in different configurations. In the AbTCR, V_L_ is fused to the TCRγ constant region and V_H_ to that of TCRδ (52). The TAC receptor fuses two scFvs with the CD4 hinge, transmembrane, and intracellular domains (53), interacting with a target cell antigen through one scFv and the ε subunit of the endogenous TCR–CD3 complex via the second scFv. The ε-TRuC receptor is formed via the incorporation of an overexpressed scFv–CD3ε fusion into the endogenous TCR–CD3 complex (54). The ε-TRuC receptor is the only of these to have been investigated for its antigen sensitivity, which showed sensitivity to be superior to a 28ζ CAR but inferior to the STAR receptor, all bearing the same antigen specificity (55). Altogether, the above studies suggest that some CD3-based receptors have superior sensitivity to canonical CARs, which increases their ability to lyse targets with low antigen densities, on the order of a few hundred in the case of HIT receptors. Importantly, the HIT receptor is the only one to replace the endogenous TCR, whereas all others generate dual-specific T cells as they retain their TCR.

### Targeting Two Antigens at Once: OR-gates

Simultaneously targeting more than one antigen is a potential remedy to prevent antigen escape, but how to do so effectively remains to be determined. One approach is to infuse multiple T cell products, each one targeting a different antigen; another is to engineer multispecific T cells.

The administration of multiple validated CAR T cells either simultaneously or sequentially is attractive for mixing and matching different cell products. Several clinical trials, mostly in B-cell malignancies, have shown the feasibility of these approaches with an acceptable safety profile (56). However, long-term responses have not improved substantially relative to single-cell product infusion, corroborating preclinical findings that predicted this outcome, especially when targeting antigens of low abundance or using less sensitive CAR designs (27, 57–59). The coinfusion of combined CAR T cell products will require further investigation of infusion dose, order, and timing and careful evaluation of synergy or dominance. These challenges support the rationale for engineering multispecific CAR T cells, especially when individual targets are of low abundance.

**Figure 2.**
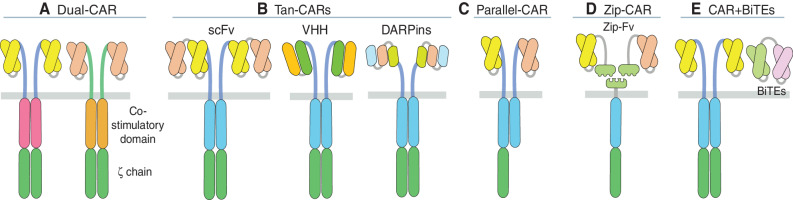
"OR" logic-gate CAR T cell designs. **A,** Dual-CAR, two distinct fully functional CARs targeting different antigens are coexpressed on the surface of the same T cell. Identical or different costimulatory domains can be used. **B,** Tan-CARs, left, bivalent single CAR chain with two binding domains (scFvs) in tandem targeting two distinct antigens. Middle, bivalent single CAR chain with two distinct binding domains (VHH, single-domain antibodies) in tandem targeting different epitopes of the same antigen. Right, multivalent single CAR chain using designed ankyrin repeat proteins (DARPIn) in tandem targeting more than two antigens. **C,** Parallel-CAR, one fully functional CAR (second generation) and a chimeric costimulatory receptor (CCR) with identical hinge domains that allow CAR-CCR heterodimerization are coexpressed on the surface of the same T cell. **D,** Zip-CAR, Zip-CAR expressed on the surface of T cells binds to distinct Zip-Fv (Zip-scFv) targeting different antigens. Zip-CAR uses a leucine zipper adapter expressed on the T cells that bind to administered Zip-Fv specific for antigens A or B. **E,** CAR-BiTEs, CAR T cell coexpressing bispecific T cell engagers (BiTEs). BiTEs are directed against CD3 and an antigen distinct from the CAR target.

The first embodiment of concomitant antigen targeting, in which either one in a pair of antigens can serve as target, is defined as an OR-gate in Boolean terminology. This goal may be achieved by constitutively coexpressing two independent CARs (hereafter referred to as Dual-CAR; Fig. 2A) or by expressing a single bivalent CAR comprising tandem binding domains (hereafter referred to as Tan-CAR; Fig. 2B). How do these approaches compare, and are there emerging rules for effective multitargeting?

#### Dual-CAR

This approach is attractive owing to the facility of coexpressing two independent, functionally validated CARs in T cells. Using a bicistronic vector encoding CD19 and CD123 BBζ CARs, Ruella and colleagues demonstrated the superiority of the Dual-CAR over pooled CD19 and CD123 CAR T cells (57). Another advantageous feature of Dual-CARs is to mix and match costimulatory domains. In leukemia models of antigen escape, dual targeting with two CARs respectively targeting CD19 and CD22 not only outperformed the sequential infusion of CD19 or CD22 monospecific CAR T cells but was superior when combining a 28ζ and a BBζ CAR as compared with coexpressing two 28ζ CARs and, even more so, two BBζ CARs. Moreover, assigning the 28ζ CAR to CD19 and the BBζ CAR to CD22 resulted in the most effective treatment (27). Shalabi and colleagues corroborated the superiority of combining 19.28ζ and 22.BBζ (60).

#### Tan-CAR

The Tan-CAR design requires careful examination of scFv order, hinge domains, and linker length to preserve efficient recognition of both antigens. Thus, cotargeting CD19 and CD22 with an optimal loop structure 19V_L_–22V_H_–22V_L_–19V_H_ to design Tan-19.22.BBζ allowed better T cell/tumor cell conjugate formation and provided higher tumor control than other variable chain orientations (58). Cotargeting CD19 with a short extracellular domain (EC) and CD20 with an extended EC (Tan-19.20 CAR) was more efficient at controlling heterogeneous tumors *in vitro* and *in vivo*, further underscoring the need to adapt Tan-CAR design to antigens topology (61). In an MM model, De Larrea and colleagues combined BCMA and GPRC5D CARs to target a heterogeneous tumor mix including a BCMA^−/−^ tumor fraction and found that pooled BBζ and Dual-BBζ CAR T cells outperformed their Tan-BCMA.GPRC5D.BBζ CAR (62). In preclinical models of glioblastoma, HER2.28ζ and IL13Rα2.28ζ Dual-CAR T cells outperformed pooled monospecific CAR T cells (63) but were later superseded by an IL13-mutein/HER2.scFv Tan-CAR that allowed for more efficient synapse formation and superior *in vivo* tumor control (64).

In a recent study, Leung and colleagues found that CD19 and CD79a cotargeting with Tan- or Dual-BBζ CARs superseded pooled monospecific CARs in preventing antigen escape (65). Despite improved tumor control *in vivo*, the Tan-CAR configuration compromised antigen binding compared with monospecific CARs, consistent with previous observations (61, 66), whereas Dual-CAR exhibited diminished signaling, possibly owing to competition for downstream signaling molecules (65). Dual-CARs that employ a common hinge/transmembrane component may heterodimerize and function as parallel CARs (see below).

Altogether, the Dual-CAR and Tan-CAR studies support the benefit of OR-gate combinatorial antigen targeting to offset antigen escape, but no single approach has proven to be uniformly superior. Antigen topology and density on tumor cells, together with CAR structural design, including binding domain affinity, hinge length, and signaling domain selection, are all parameters that require careful optimization.

#### Dual-Antigen Targeting in the Clinic

Several Dual-CAR and Tan-CAR T cells are already in the clinic in the setting of B-cell malignancies and MM (Table 2), with more to begin clinical investigation in solid tumors (56). Although published clinical trials are still few and lack single-target comparisons, the feasibility and safety profile of dual-targeting are encouraging, even though early results have not proven so far to be superior to historical controls obtained with single-antigen targeting (Tables 1 and 2; refs. 56, 67, 68). Despite its superiority to pooled and Dual-CARs in preclinical models, Tan-19.22.BBζ CAR treatment has still resulted in disease relapses, which have been pinned on suboptimal CD22 responses (58, 60, 66). The single configuration of BBζ may not be best suited to target tumors with low abundant antigen densities, particularly in patients who manifested a low level of CD19 after previous axicabtagene ciloleucel treatment. Shalabi and colleagues, thus, proposed to adopt dual 19.28ζ and 22.BBζ for future clinical trials (27, 60). Tumor relapses have also occurred following Tan-19.20 CAR treatment in B-cell lymphoma, although owing to poor *in vivo* T cell expansion rather than antigen downregulation (69).

**Table 2.** Clinical trials investigating bispecific CAR T cells in B-cell malignancies and MM

Antigen target	CAR design		Disease	CR (*n* = treated patients)	References
**CD19 + CD22**	**Tandem**	19.22.4-1BBζ	B-ALL	87% (*n* = 15)	Wang Y et al., 2020
			B-ALL	100% (*n* = 6)	Dai H et al., 2020
			B-ALL	88% (*n* = 17)	Spiegel J et al., 2021
			DLBCL	29% (*n* = 21)	Spiegel J et al., 2021
			DLBCL	63% (*n* = 16)	Wei G et al., 2021
			B-ALL	83% (*n* = 7)	Hu Y et al., 2021
			DLBCL	64% (*n* = 33)	Qu C et al., 2022
			B-ALL	60% (*n* = 20)	Shalabi H et al., 2022
	**Dual**	19.OX40ζ and 22.4-1BBζ	B-ALL	86% (*n* = 15)	Cordoba S et al., 2021
**CD19 + CD20**	**Tandem**	20.19.4-1BBζ	NHL	63% (*n* = 19)	Shah N et al., 2020
			CLL	67% (*n* = 3)	Shah N et al., 2020
			NHL	70% (*n* = 87)	Zhang Y et al., 2022
			NHL	70% (*n* = 10)	Larson SM et al., 2023
**BCMA + CD38**	**Tandem**	BCMA.38.4-1BBζ	MM	52% (*n* = 23)*	Mei H et al., 2021
**BCMA**	**Biepitope**	BCMA.4-1BBζ	MM	77% (*n* = 17)*	Xu J et al., 2019

Abbreviations: CR, complete response; DLBCL, diffuse large B-cell lymphoma. *Stringent CR.

In ALL patients, Cordoba and colleagues evaluated Dual-CAR T cells expressing a 19.OX40ζ CAR and a novel 22.BBζ CAR using a pentameric coiled-coil hinge domain to increase sensitivity to CD22 (70). Despite encouraging initial remissions, 70% of the patients relapsed within a year of the treatment, attributed mainly to lack of T cell persistence but also CD19 loss.

Interestingly, the biepitope targeting of BCMA via two tandem VHH elements (Fig. 2B) and Tan.BCMA.CD38 CAR has shown high complete response rates (Table 2; refs. 15, 71), leading to the former's rapid FDA approval for adults with relapsed or refractory MM (Tables 1 and 2). Relapses occurring in both clinical trials have been attributed to insufficient T cell persistence (15, 71).

Altogether, these early trials on dual-targeted T cells in hematologic malignancies are encouraging but highlight the need to stringently optimize antigen binding and CAR signaling to achieve the required antigen sensitivity while providing a sustained tumor response.

#### Other OR-gate Entities

Engineering trivalent CAR T cells has been achieved using tricistronic vectors to target HER2.IL13Ra2.Epha2 or CD19.CD22.CD20 (72–74). From a technical standpoint, large vector inserts encumber efficient gene delivery, whereas cotransduction of single CAR vectors generates T cell mixtures with variegated specificity and an increased risk of insertional mutagenesis. Polypeptides that bind antigens with high affinity, known as designed ankyrin repeat proteins (DARPin), have been successfully employed instead of scFvs to generate less bulky trispecific CARs targeting EGFR, EpCAM, and HER2 (Fig. 2B; ref. 75). In another strategy, ligands that bind to several receptors on tumor cells can be incorporated into CARs, as recently shown using a BAFF–ligand CAR, which engages BAFFR, TACI, and BCMA, although the efficacy of this design compared with conventional scFv-based CARs is still unknown (76).

Another OR-gate design is based on the heterodimerization of two chains, wherein a CAR chain specific for one antigen dimerizes with a chimeric costimulatory receptor (CCR) chain specific for a second antigen facilitated by the intentional choice of the same H/TM domains for each receptor (Fig. 2C). Thus, MUC-1.28ζ+ERbb2.28, GD2.28ζ+B7H3.BB, or MSLN.28ζ+CSPG4.BB provided optimal tumor-dependent costimulation and enhanced tumor control in several models of solid tumors, further supporting the benefit of providing both CD28 and 4-1BB support to engineered T cells (77, 78).

Modular Zip-CAR systems that utilize a leucine zipper adapter expressed on the T cells to bind to Zip-Fv (Zip-scFv) offer a versatile OR-gate tool that can be adapted to several antigens, whereas the dose and binding strength of Zip-Fv can be tuned to regulate T cell activity or can be terminated by a competitive nonantigen-binding Zip-Fv (Fig. 2D; refs. 79, 80). Multiantigen targeting can also be achieved by engineering CAR T cells to secrete a dual-engager protein [e.g., EGFRvIII-CAR and EGFR-bispecific T cell engager (BiTE); Fig. 2E; ref. 81].

Altogether, a rapidly expanding number of studies demonstrate the potential of OR-gates to broaden antigen coverage and mitigate antigen escape. However, how to effectively achieve multiantigen recognition and ensure functional CAR T cell persistence has not yet been established. Moreover, the repurposing of an intact TCR–CD3 complex to increase antigen sensitivity and the targeting of multiple antigens via an OR-gate may increase the risk of on-target/off-tumor toxicity, thus requiring careful antigen selection, especially in solid tumors in which target antigens are often shared with normal tissues, and suitable preclinical modeling.

## LOGIC GATING TO AUGMENT CAR T CELL SELECTIVITY AND SAFETY

CD19 was selected as a CAR target in part for its limited expression in normal cells (5), which is largely confined to B cells and thus exposes CD19 CAR T cell recipients to B-cell aplasia, a clinically manageable on-target/off-tumor toxicity. Targeting CD20, CD22, and BCMA likewise exposes recipients to restricted on-target tissue damage. However, many potential CAR targets, especially in solid tumors, are expressed in cell types that cannot sustain an immune assault, calling for the need to detarget those cell types. Short of identifying tumor-specific epitopes, one needs to design T cells that preferentially engage tumor cells and spare normal cells. Differential levels of expression alone provide, in some instances, a therapeutic window that can be exploited to spare low-antigen-expressing normal cells based on CAR antigen sensitivity alone. T cell engineering, however, opens many more opportunities for discerning malignant from normal cells. An early embodiment of this concept was to design two antigen-reactive receptors, respectively, specific for antigens A and B that alone did not lyse normal cells expressing either A or B but in concert killed tumor cells that expressed both A and B (82).

**Figure 3.**
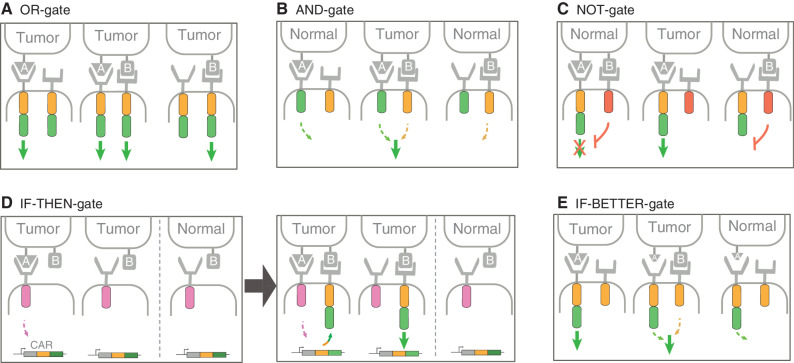
Principles of logic-gated CAR T cells. **A,** OR-gate, T cells coexpress fully functional CARs targeting distinct tumor antigens A and B. **B,** AND-gate, T cells coexpress a CAR specific for antigen A and a CCR specific for antigen B. CAR T cells are fully activated when the CAR and the CCR simultaneously engage with antigens A and B coexpressed on the tumor but not on normal cells. **C,** NOT-gate, T cells coexpress a fully functional CAR specific for antigen A and an inhibitory CAR (iCAR) specific for antigen B. T cells are fully activated when the CAR engages with antigen A expressed exclusively on tumor cells. iCAR engagement with antigen B expressed on normal cells reversibly inhibits CAR T cells. **D,** IF-THEN-gate, T cells coexpress synthetic Notch (SynNotch) receptor specific for antigen A. Engagement of SynNotch receptor with antigen A (left) induces transient expression of fully functional CAR specific for antigen B (right) in the tumor environment. The decay of CAR expression (spatiotemporal regulation) in circulating T cells should allow for the protection of normal cells expressing the tumor-associated antigen B. The gray arrow between implies time. **E,** IF-BETTER-gate, T cells coexpress a fully functional CAR specific for antigen A and a CCR specific for antigen B. CAR T cells are fully activated if the CAR engages with antigen A expressed at high levels on tumor cells. If tumors express antigen A at low levels (small-size antigen A, middle tumor cell), full T cell activation requires CCR engagement with antigen B on the same tumor cells. Antigen A can be expressed alone, not with antigen B, at low levels in normal tissues.

Such combinatorial strategies to design discriminatory, tumor-selective T cells are now flourishing and can be classified using Boolean terminology (Fig. 3). We focus here on strategies that require combinatorial antigen input to direct T cell activity. The OR-gate strategies (Fig. 3A) reviewed above are only one of several ways to gate tumor recognition. Other approaches to manage T cell specificity and limit on-target toxicities such as remote controls, suicide genes, or binding domain affinity tuning are reviewed elsewhere (83–85).

### AND-gates

One tumor-targeting concept that aims to create tumor specificity from a set of nonspecific targets is based on the Boolean AND-gate (Fig. 3B). In this instance, productive T cell activation depends on combined inputs emanating from two antigens coexpressed in the tumor but not in normal cells (the latter may express either one alone). Each separate input must therefore not suffice to trigger target cell lysis but exceed an activation threshold upon their simultaneous engagement. One early embodiment was exemplified by Kloss and colleagues, who combined a defective ζ chain–based CAR specific for prostate-specific membrane antigen (PSMA) with a CCR with CD28 and 4-1BB endodomains to rescue the poor activation signal provided by the CAR (82). A related design combining a first-generation CAR lacking a costimulatory endodomain with a CCR providing costimulation has been touted as an AND-gate (86–88) but rather functions as an IF-BETTER-gate (see below). Under certain conditions, IF-BETTER- and OR-gates may operate as an AND-gate, but an authentic AND-gate requires minimal or absent T cell activation in the presence of either target alone.

Tousley and colleagues recently described the Logic-gated Intracellular NetworK CAR (LINK-CAR), wherein the split CD3ζ and costimulatory domains are replaced with LAT and SLP-76 molecules. To optimize LINK-CAR specificity, point mutations were introduced into LAT (del171–233) and SLP76 (del224–244) to minimize the engagement of the adapter protein GADS essential for LAT and SLP76 interaction. In a proof-of-concept model targeting CD19 and ROR1 in NALM6 cells, only the optimized LINK-CAR achieved tumor control in the absence of ROR1 on-target/off-tumor toxicities in a mouse model. In this study, the LINK-CAR was less toxic than a CD19-synthetic Notch receptor (SynNotch)→ ROR1-CAR design (see IF-THEN-gate, below), whereas the split CAR (ROR-1.ζ and CD19.28) was ineffective (89).

AND-gates might be achieved using modular Zip-CAR strategies if CD3ζ and CCR modules can be suitably affinity-tuned (80), but their *in vivo* safety and efficacy remain to be determined. Stringent AND-gates are challenging to achieve and have not yet been tested clinically, but their potential to unleash T cell potency limited to tumor cells is attractive.

### NOT-gates

This form of gating depends on an inhibitory CAR (iCAR) to turn off CAR T cell activity upon encountering unintended target cells. In this instance, antigen A on tumor cells and normal cells is targeted by an activating CAR but is impeded upon engaging antigen B that is present on the normal cells only (Fig. 3C). As a proof of concept, Fedorov and colleagues engineered a PSMA.iCAR incorporating the endodomain of the inhibitory molecule PD-1 to reversibly restrict CD19 CAR T cell activity against CD19^+^ PSMA^+^ cells without interfering with antitumor CD19 CAR activity against CD19^+^PSMA^−^ cells (90). PD-1–based iCARs also abated TCR-mediated allogeneic response with an iCAR directed to HLA molecules (90). In another example, Richards and colleagues combined CD93 targeting with a CD19.iCAR based on PD-1 or TIGIT inhibitory domains to spare CD93^+^CD19^+^ cells (91). Exploiting the frequent loss of HLA in some cancer cells, NOT-gates utilizing a PD-1–based iCAR against HLA-A*02:01 or A*03:01 have been paired with a 28ζ CAR targeting a different HLA allele retained by tumors (92). A related approach has been devised to selectively target CEA^+^/HLA^−^ tumor cells and spare HLA^+^ cells using the inhibitory leukocyte Ig-like receptor 1 (LIR1) that binds to the HLA-A*02 molecule (93).

Much like AND-gate, the level of expression of the CARs and the antigen pairing are critical for optimal NOT-gate function, which depends on a balance between activation and inhibition strength of signaling (90). Future studies using tuned receptor affinity, balanced CAR and iCAR signaling, and optimal target selection may allow for the translation of NOT-gates to the clinical setting.

### IF-THEN-gates

Spatiotemporal regulation of CAR expression is an attractive concept to restrict a conditionally expressed CAR that poses safety concerns. In this instance, engagement of antigen A by a CAR or another sensor triggers transient expression of a CAR specific for antigen B, thus restricting B CAR expression to the tumor (space) but not beyond when (time) the T cell reaches a normal tissue (Fig. 3D). The Lim lab combined the use of a cleavable Notch receptor with the induction of CAR expression under the control of a synthetic transcription factor (TF) released upon cleavage of SynNotch (94, 95). The released TF then binds to a responsive promoter located upstream of a specific transgene such as CARs, thus making this system an IF-THEN-gate. Using such an on-off switch circuit, the authors showed that CAR expression is only induced upon activation of the SynNotch receptor leading to the eradication of tumors coexpressing the SynNotch and CAR ligands (96, 97). This approach has since been widely applied to a range of preclinical mouse models to treat, for example, mesothelioma coexpressing ALPPL2 and MCAM antigens (ALPPL2-SynNotch and MCAM-CAR) or glioblastoma expressing either EphA2 or IL13Ra2 present in the vicinity of EGFRvIII^+^ tumor cells (SynNotch-EGFRvIII and EphA2.IL13Ra2 Tan-CAR; refs. 98, 99). In these models, SynNotch-regulated CAR expression outperformed single CAR strategies by limiting tonic signaling leading to less exhausted and long-lived memory T cells (98, 99). Hernandez-Lopez and colleagues further harnessed the SynNotch system to increase specificity for HER2^+^ tumors devising a low-affinity HER2-SynNotch that gated expression of a high-affinity HER2 CAR, showing effective *in vivo* induction by tumor cells expressing HER2 in the range of 10^7^ molecules per cell but not 60,000 molecules per cell (100). The effectiveness of this sensing system at low antigen densities remains to be evaluated.

As mouse SynNotch and synthetic TFs may be immunogenic, Zhu and colleagues described humanized SynNotch-like receptors named SyNthetic Intramembrane Proteolysis Receptors (SNIPR) by assembling a library of human EC, transmembrane (TMD), and juxtamembrane domains (JMD) that allow for ligand-dependent release of orthogonal synthetic TF to deliver therapeutic payloads such as IL2 or CARs. Combination of human CD8α-EC, Notch-TMD/JMD, and HNF1A (DNA-binding domains) fused to the transactivation domain of human NF-κB p65 induced CAR expression similarly to conventional SynNotch, successfully eradicating tumors expressing antigens in preclinical animal models (101). The SynNotch system can also be adapted to function as a NOT-gate (OFF-Notch) by inducing proapoptotic factor tBID (truncated BH3-interacting domain death agonist) upon off-tumor recognition, leading to rapid T cell death. Although this system curbs toxicity, it functions similarly to suicide genes or antibody-mediated depletion leading to T cell loss, which also curtails therapeutic efficacy (102).

Antigen selection for IF-THEN gating requires careful pairing, as the kinetics of CAR induction and decay play a critical role in avoiding on-target/off-tumor toxicities. Srivastava and colleagues found that EPCAM-SynNotch–regulated ROR1-CAR T cells failed to protect ROR1^+^ normal stroma cells when tumors were in close proximity or disseminated (103). In a different antigen pairing, controlling ROR1-CAR with SynNotch B7H3 (B7H3 is absent on normal ROR1^+^ stroma cells) was efficient at mitigating on-target/off-tumor toxicities (103). No SynNotch system is yet in the clinic, but the recent development of humanized systems and further kinetic optimizations are poised to accelerate the clinical application of this technology.

### IF-BETTER-gates

An IF-BETTER-gate is one in which a CAR-engaging antigen A performs better in the presence of antigen B. B is not obligate for CAR T cell function (for example, if A is abundant) but the presence of B helps activation following recognition of A when the latter's abundance is limiting (Fig. 3E). IF-BETTER thus differs from the obligate dual-requirement for A and B in an AND-gate and from the temporally regulated IF-THEN-gate. Recognition of B is not mediated by an activating receptor and may consist in a CCR or simply a cell-surface-anchored scFv. It, therefore, does not incur the potential on-target/off-tumor toxicity imparted by combining a second CAR, as is done in an OR-gate. Wilkie and colleagues coexpressed a ζ chain–based CAR (HER2.ζ) and a MUC1.28 CCR to target HER2-high but not HER2-low cell lines. Complementary signaling from CAR and CCR upon dual-antigen recognition showed enhanced *in vitro* cytokine secretion and T cell proliferation (88). A similar concept was applied by combining a mesothelin CAR and a Folate receptor CCR to target ovarian cancer or combining a CD13 CAR and a TIM3 CCR to target acute myeloid leukemia (AML). With the CAR alone, tumors were transiently controlled *in vivo*, in contrast to the conditions where CAR and CCR antigens are present (83, 84). Katsarou and colleagues combined a fully functional 28ζ CAR targeting either BCMA or CD19 with a CCR binding to CD38, an antigen highly expressed in B-cell malignancies but also present in normal immune cells including T cells (44). In order to not direct cytolysis to cells expressing antigen B, the CAR and CCR used to create an IF-BETTER-gate must differ structurally so as to not allow heterodimerization as in the parallel CAR design (77, 78). Use of a CCR rather than a cell-bound scFv supports CAR signaling and T cell persistence through its costimulatory function, which should be selected to complement the paired CAR (44). Thus, combining a CD19 or BCMA.28ζ CAR with a high-affinity CD38 CCR increased cytokine production, T cell persistence, and *in vivo* control of tumors, including CD19^+^ leukemia with <1,000 molecules per cell (44).

IF-BETTER-gates yield a CAR T cell that can modulate antigen sensitivity to A in the presence of B and thus imparts T cell “preference” for cells expressing B, but with less stringency than an AND-gate and allowing broader target selection for B than an OR-gate. IF-BETTER-gates have only been reported for preclinical B-cell malignancies and myeloma models but may be especially useful for AML and solid tumors, for which highly expressed antigens with restricted systemic expression are scarce.

Gating strategies that aim to augment CAR T cell tumor selectivity are a promising approach to mitigate unwanted on-target/off-tumor toxicities. These strategies are often initially tested in proof-of-principle models wherein tumors express abundant antigen levels and animals lack faithful tumor microenvironments. Defining clinically relevant antigen pairs and realistic parameters for their targeting (e.g., level of antigen expression on tumors vs. normal tissues, scFv affinity, tumor accessibility and tumor environment), particularly in solid tumors, are important follow-up studies to guide successful clinical development.

## PRODUCTION OF ANTIGEN-SENSITIVE AND DUAL-TARGETED CAR T CELLS

The successful introduction of more complex T cell engineering strategies in the clinic will also depend on advances in T cell manufacturing. At present, CAR T cells are most often manufactured using viral vectors (21). After chromosomal integration, CAR expression is driven by the 5′ long terminal repeat in γ-retroviral vectors or by an exogenous promoter, usually long EF1α, in lentiviral vectors. Although CAR expression is variegated in T cells due to the semirandom integration pattern (104), CAR T cells have shown remarkable therapeutic activity against hematologic malignancies using either γ-retroviral or lentiviral vectors (Table 1). Nonviral approaches have also been used to generate clinical-grade CAR T cells, primarily utilizing the Sleeping Beauty or piggyBac transposons (105, 106). Here, T cells are electroporated with two plasmids, one encoding a transposase and the other the transposon that encodes the CAR, also resulting in variegated CAR expression owing to the semirandom integration of transposon DNA (107).

**Figure 4.**
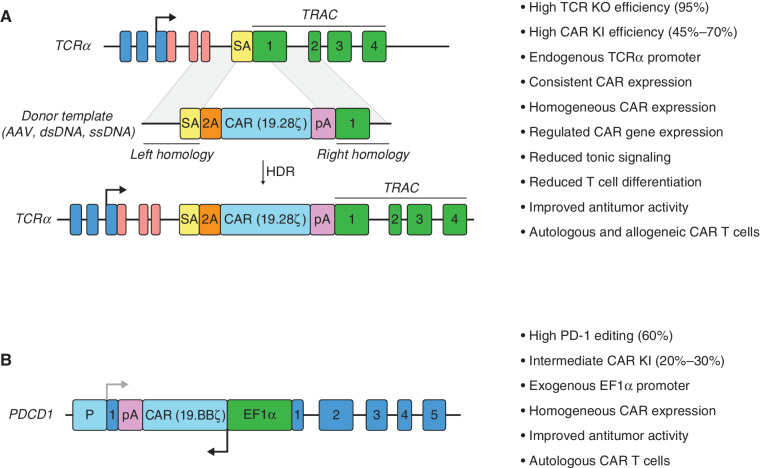
Site-specific integration of CAR cDNA in T cells. **A,** In *TRAC*-CAR T cells, the CAR gene is inserted upstream of the *TRAC* exon 1, and it is flanked by splicing acceptor (SA) and 2A sequences to the 5′ end and polyadenylation (pA) sequence to the 3′ end. CAR expression is controlled by the endogenous TCRα promoter. This strategy also leads to the disruption of endogenous TCRα expression and consequently to the disruption of the TCR–CD3 complex surface expression. A number of other advantageous features are highlighted to the right. AAV, adeno-associated viruses; dsDNA, double-stranded DNA; HDR, homology-directed repair; KI, knockin; ssDNA, single-stranded DNA. **B,** In *PDCD1*-EF1α-CAR T cells, an EF1α-CAR-pA transcription unit is inserted in the exon 1 of the *PDCD1* locus in an orientation opposite to *PDCD1* transcription directionality. This strategy shares some features with the *TRAC*-CAR approach, indicated at the right, but there also are clear distinctions.

The benefit of tightly controlling CAR expression has been demonstrated by targeting the CAR cDNA to the TCR alpha constant (*TRAC*) locus using sequence-specific chimeric nucleases (108). Integration of the 19.28ζ cDNA in exon 1 of the *TRAC* locus improved antitumor T cell activity relative to retroviral-encoded CAR, owing to reduced tonic signaling and delayed T cell differentiation upon repeated antigen stimulation (Fig. 4A; ref. 108). *TRAC*-CAR T cells that control CAR expression from the endogenous TCRα promoter show homogeneous and consistent CAR expression levels across multiple T cell donors, which contrasts with virally modified CAR T cells (108). Moreover, due to high TCR knockout (KO) efficiency, *TRAC*-CAR T cells could be used in both autologous and allogeneic settings. The superior antitumor activity of *TRAC*-CAR versus retroviral CAR T cells has also been shown in a syngeneic mouse model (109). Alternatively, CAR cDNAs have also been integrated at other loci under control of either endogenous or exogenous promoters. Zhang and colleagues further found that CAR T cells bearing an EF1α-controlled 19.BBζ CAR transcription unit integrated into the *PDCD1* exon 1 show improved antitumor activity compared with lentivirally modified CAR T cells (Fig. 4B; ref. 110). Another key feature of this design is the reduction of PD-1 expression, which may decrease CAR T cell exhaustion and enhance functional T cell persistence. However, not all integrated CAR transcription units lead to optimal antitumor activity (108, 111, 112), requiring careful evaluation of promoter selection at any given chromosomal site.

T cells expressing CD3 complex–based receptors have been engineered using site-specific integration (HIT receptor) or lentiviral/γ-retroviral vectors (STAR, TCAR, AbTCR, ε-TRuC, and TAC receptors). To engineer HIT T cells, we relied on our *TRAC*-CAR strategy (108) to insert the V_H_–Cβ–P2A–V_L_–*TRAC* exon1 donor sequence to express the chimeric V_H_–Cβ and V_L_–Cα chains under the control of the endogenous TCRα promoter (36). Importantly, this strategy also results in the elimination of the endogenous αβ TCR, thus abolishing the potential alloreactivity of HIT T cells and the competition between chimeric and endogenous TCRs. As for STAR, TCAR, and AbTCR T cells, the chimeric receptors are expressed by using lentiviral/γ-retroviral vectors and thus show variegated expression (49, 50, 52). To circumvent genetic disruption of the endogenous αβ TCR, these strategies made use of different C domains to minimize TCR chain mispairing. The STAR receptor contains mutated mouse Cα and Cβ regions (49), the AbTCR receptor contains human Cγ and Cδ domains (52), and the TCAR receptor contains human Cα and Cβ regions fused to V domains in tandem (V_H_–V_H_–Cα and V_L_–V_L_–Cβ; Fig. 1B; ref. 50). These modifications do not eliminate the interaction of the endogenous αβ TCR and CD3 complex, which is needed to assemble STAR, TCAR, and AbTCR receptors at the surface; this also leads to the retention of their potential alloreactivity. In ε-TRuC and TAC T cells, the scFv–CD3ε and scFv1–scFv2–CD4 fusions, respectively, are overexpressed using lentiviral vectors (53, 54). These fusions do not contain TCR elements and therefore depend on expression of the endogenous αβ TCR to properly assemble at the surface. This results in ε-TRuC and TAC T cells possessing dual specificity and potential alloreactivity as well.

Many of the above studies underscore the broad potential of site-specific genome engineering to develop improved CAR T cells for a variety of purposes (113, 114). For example, *TRAC* or *TRBC* KO eliminates alloreactivity of CAR T cells (115); *PDCD1* KO may reduce T cell exhaustion (116); *CD52* KO eases the use of *TRAC* KO-CAR T cells for allogeneic applications in combination with anti-CD52 antibodies to deplete host T cells (117–119). However, gene editing approaches can lead to genetic abnormalities in T cells. Triple CRISPR/Cas9-mediated KO ablating *TRAC*, *TRBC*, and *PDCD1* can induce frequent chromosomal translocations (120). Translocations have also been detected when targeting *TRAC* and *CD52* loci with either TALEN or CRISPR/Cas9 (119, 121), and aneuploidy when editing three loci with CRISPR/Cas9 RNPs (122). These observations put a note of caution when multiplexing double-strand breaks and increase interest in alternative DNA editing methods based on CRISPR/Cas9 nickases, such as base editing and prime editing (123).

Development of CAR T cells with additional functionalities will require more complex genetic modifications. Though γ-retroviral and lentiviral are sufficient to deliver AND, OR, IF-THEN, and IF-BETTER gate constructs, and other simple genetic constructs, their limited cargo capacity will constrain delivery of more sophisticated circuits. Transposon-mediated delivery could potentially address this limitation. However, approaches requiring homogeneous gene expression may not be advanced by this method. PASTE technology enables integration of large cargos (up to ∼36 kb) into specific loci in human cells, including primary T cells. The platform uses a CRISPR/Cas9 nickase fused to both a reverse transcriptase and a serine integrase, a pegRNA, and a minicircle cargo plasmid. The pegRNA contains the serine-integrase site, which is inserted into the target DNA via prime editing; then the serine integrase introduces the cargo plasmid at the integrase site (124).

Another important variable for effective T cell engineering is the selection of loci or extragenic chromosomal regions where not only CAR genes but more complex genetic circuits could be integrated and reliably expressed (125). We recently identified an extragenic region on human chromosome 7, termed GSH6, that supports CAR expression as effectively as the *TRAC* locus (126). With these novel gene editing/targeting tools to target gene loci or extragenic genomic safe harbors, one can expect rapid progress in engineering T cells endowed with precisely calibrated functions to achieve greater T cell specificity and potency.

## ADVANCES IN T CELL MANUFACTURING

Integrating next-generation CAR T cell designs targeting multiple antigens together with the progress in understanding the basis for T cell differentiation states at the transcriptional and epigenetic levels should inform improved methods to generate better autologous and allogeneic CAR T cells. In concert with fine-tuning combinatorial receptor signaling, novel manufacturing processes can be adopted to modulate transcriptional, epigenetic, and metabolic pathways.

From a production standpoint, Dual- and Tan-CARs expressed from a single vector maintain the cost-effectiveness and relative ease of manufacturing of single-CAR T cell products, averting the need to produce two cell products or two separate vectors and ensuing product heterogeneity. Cell dosing in Tan-CAR T trials tends to mimic those dispensed in single-CAR T trials (15, 60, 66, 69, 70, 127). New CAR designs (37, 108) and rapid manufacturing platforms (128), however, offer the prospect of lowering effective cell doses. For example, therapeutic doses as low as 25 × 10^6^ autologous CAR T cells were recently reported by Park and colleagues in adult patients with relapsed/refractory (R/R) diffuse large B-cell lymphoma (NCT04464200), using a human CD19–targeted calibrated 19.28ζ-1xx (37). The dose-expansion phase of this trial is now proceeding with only 25 × 10^6^ CAR T cells per infusion (129). Svoboda and colleagues reported therapeutic doses in the range of 3 × 10^6^ to 30 × 10^6^ CAR T cells in patients with R/R NHL (NCT04684563) using IL18 secreting autologous 19.BBζ CAR T cells (huCART19-IL18; ref. 130). The FasT CAR-T (F-CAR-T) manufacturing platform in which T cell culture time is less than 24 hours was evaluated in two phase I clinical studies in patients with B-ALL using either CD19 or 19.22 Dual-CAR T cells (NCT03825718 and NCT04129099). F-CAR T cells were successfully manufactured, and cell doses as low as 0.3 × 10^5^ to 1.5 × 10^5^ CAR^+^ T cells/kg enriched in stem cell memory T (T_SCM_) and central memory T (T_CM_) cells as shown in preclinical studies were infused in 25 patients (NCT03825718). The safety of this approach, its neurotoxicity in particular, as well as its efficacy, will need to be further evaluated, as most responders to CD19 F-CAR T therapy subsequently received allogeneic hematopoietic stem cell transplantation therapy (131, 132). The ability to rely on low cell infusion doses raises the prospect that blood draws rather than leukapheresis products may be sufficient to manufacture autologous T cell products. Increased T cell potency and shorter culture times that require reduced amounts of consumables, reagents, and manpower bestow logistics and financial advantages that could broaden patient access, provided that release testing with adequate sensitivity can be adapted to processes necessitating a low number of cells.

CAR T cells derived from naive T cells as well as CAR T cells endowed with T_CM_ and T_SCM_ phenotypes may further increase antitumor efficacy by providing greater functional persistence. Biasco and colleagues analyzed CAR T cell phenotypes in pre- and postinfusion samples and established the critical role of T_SCM_ in mediating early antileukemic responses and long-term persistence of CAR T cells (133). Enriching for T_CM_ cells and T_SCM_ cells prior to *ex vivo* expansion or adoptive transfer can also improve high frequencies of persistent cells with stem cell–like characteristics. Arcangeli and colleagues have shown that CAR T cell manufacturing from naive/stem memory T lymphocytes enhances *in vivo* antitumor responses in a leukemia-bearing humanized mouse model while curtailing cytokine release syndrome (134). Cytokines such as IL7, IL15, and IL21, small-molecule treatments, and antioxidants such as N-acetylcysteine (NAC) have the potential to enhance the *ex vivo* maintenance of T_CM_ and T_SCM_ cell subsets and to enable the expansion of more potent antitumor T_SCM_ cells (NCT04464200; refs. 129, 135). Further studies will be required to evaluate the relevance and functionality of T_SCM_ cells in patients with cancer as well as the optimal conditions for their manipulation.

Building on the approval by the FDA of six CAR T cell therapies since 2017, manufacturing platforms are evolving toward closed and automated systems (recently reviewed in ref. 127) in order to bolster reproducibility and patient access. Control of T cell activation is key. Interestingly, Shalabi and colleagues (60) and Spiegel and colleagues (66) reported that earlier removal of Transact beads during *ex vivo* expansion shortens the time to reach the cell dose, suggesting that prolonged CD3/CD28 activation is detrimental. In addition, Ghassemi and colleagues have recently shown that this step is dispensable in a short manufacturing protocol (128).

Cryopreservation enables the storage and distribution of drug products. The efficacy of cryopreserved CAR T cells was demonstrated to be comparable to fresh CAR T cells upon measuring *in vivo* expansion, persistence, incidence of toxicities, and disease response (136). Based on this study and data from two clinical trials (NCT02315612 and NCT03448393), CAR T cells can be cryopreserved without altering their functionality, providing greater flexibility for scheduling infusions and delivery to CAR T cell administration sites.

Cargo delivery through viral vectors can be complemented or replaced by gene editing platforms. In a clinical trial for R/R B-cell NHL, Zhang and colleagues used a homology-directed repair template in the form of a linear dsDNA containing the CD19 CAR to target the CAR cDNA to the *PDCD1* locus (Fig. 4), achieving a high rate of complete remission (87.5%) and durable responses without serious adverse events (ref. 110; NCT04213469). Gene editing is attractive in the context of multiplexed genetic modifications involving KO and knockin. Clonal screening following induced pluripotency reprogramming and multiplexed editing allows for selecting safe T cell reservoirs without translocations, aneuploidy, or mutations in addition to enabling mass production of CAR T cells (137). Finally, the emergence of *in vivo* T cell engineering opens a new set of possibilities and challenges. Polymer or lipid nanoparticles, retroviral particles, and redirected viral vectors derived from HIV-1 are being tested for the *in vivo* generation of CAR T cells. Their clinical application will require optimized delivery and close monitoring of off-target effects (138–140).

## CONCLUSION AND PERSPECTIVES

The CD19 CAR therapy paradigm has spawned a torrent of CAR T cell innovation with potential applications in virtually any cancer. These endeavors will need to overcome the common challenge of antigen expression heterogeneity. Furthermore, many attractive tumor targets may be found in indispensable, normal cell types, calling for the need to maximize tumor specificity to minimize collateral on-target toxicities. New chimeric receptor designs are poised to improve CAR T cell efficacy against tumor cells expressing <1,000 target molecules per cell. Several strategies are emerging to increase tumor specificity and safety based on dual-antigen targeting and logic gating (OR, AND, NOT, IF-THEN, and IF-BETTER gates), which may be further combined (e.g., OR-NOT-gate). The targeting of more than one antigen may be achieved through multispecific CARs, coexpressed CARs, or reversibly targetable CARs. The first Tan- and Dual-CAR clinical trials in B-cell malignancies, however, illustrate the complexity of multitargeting. In solid tumors, multitargeting is likely to be critical as well and will require the development of more probing preclinical models. Although genome editing is useful to expand the realm of T cell engineering, the risks of genomic abnormalities following induced double-strand breaks should not be underestimated. Finally, although the horizons for T cell engineering will further expand with the emergence of allogeneic approaches, induced pluripotent stem cells, and *in situ* engineering, autologous manufacturing remains the cornerstone of current clinical exploration.

## References

[1] June CH , Sadelain M. Chimeric antigen receptor therapy. N Engl J Med 2018;379:64–73. 29972754 10.1056/NEJMra1706169 PMC7433347

[2] Sadelain M , Riviere I, Riddell S. Therapeutic T cell engineering. Nature 2017;545:423–31. 28541315 10.1038/nature22395 PMC5632949

[3] van der Stegen SJ , Hamieh M, Sadelain M. The pharmacology of second-generation chimeric antigen receptors. Nat Rev Drug Discov 2015;14:499–509. 26129802 10.1038/nrd4597 PMC6410718

[4] Guedan S , Ruella M, June CH. Emerging cellular therapies for cancer. Annu Rev Immunol 2019;37:145–71. 30526160 10.1146/annurev-immunol-042718-041407 PMC7399614

[5] Brentjens RJ , Latouche JB, Santos E, Marti F, Gong MC, Lyddane C, . Eradication of systemic B-cell tumors by genetically targeted human T lymphocytes co-stimulated by CD80 and interleukin-15. Nat Med 2003;9:279–86. 12579196 10.1038/nm827

[6] Brentjens RJ , Riviere I, Park JH, Davila ML, Wang X, Stefanski J, . Safety and persistence of adoptively transferred autologous CD19-targeted T cells in patients with relapsed or chemotherapy refractory B-cell leukemias. Blood 2011;118:4817–28. 21849486 10.1182/blood-2011-04-348540 PMC3208293

[7] Kalos M , Levine BL, Porter DL, Katz S, Grupp SA, Bagg A, . T cells with chimeric antigen receptors have potent antitumor effects and can establish memory in patients with advanced leukemia. Sci Transl Med 2011;3:95ra73. 10.1126/scitranslmed.3002842 PMC3393096 21832238

[8] Brentjens RJ , Davila ML, Riviere I, Park J, Wang X, Cowell LG, . CD19-targeted T cells rapidly induce molecular remissions in adults with chemotherapy-refractory acute lymphoblastic leukemia. Sci Transl Med 2013;5:177ra38. 10.1126/scitranslmed.3005930 PMC3742551 23515080

[9] Lee DW , Kochenderfer JN, Stetler-Stevenson M, Cui YK, Delbrook C, Feldman SA, . T cells expressing CD19 chimeric antigen receptors for acute lymphoblastic leukaemia in children and young adults: a phase 1 dose-escalation trial. Lancet 2015;385:517–28. 25319501 10.1016/S0140-6736(14)61403-3 PMC7065359

[10] Maude SL , Frey N, Shaw PA, Aplenc R, Barrett DM, Bunin NJ, . Chimeric antigen receptor T cells for sustained remissions in leukemia. N Engl J Med 2014;371:1507–17. 25317870 10.1056/NEJMoa1407222 PMC4267531

[11] Turtle CJ , Hanafi LA, Berger C, Gooley TA, Cherian S, Hudecek M, . CD19 CAR-T cells of defined CD4+:CD8+ composition in adult B cell ALL patients. J Clin Invest 2016;126:2123–38. 27111235 10.1172/JCI85309 PMC4887159

[12] Kochenderfer JN , Dudley ME, Feldman SA, Wilson WH, Spaner DE, Maric I, . B-cell depletion and remissions of malignancy along with cytokine-associated toxicity in a clinical trial of anti-CD19 chimeric-antigen-receptor-transduced T cells. Blood 2012;119:2709–20. 22160384 10.1182/blood-2011-10-384388 PMC3327450

[13] Brudno JN , Maric I, Hartman SD, Rose JJ, Wang M, Lam N, . T cells genetically modified to express an anti-B-cell maturation antigen chimeric antigen receptor cause remissions of poor-prognosis relapsed multiple myeloma. J Clin Oncol 2018;36:2267–80. 29812997 10.1200/JCO.2018.77.8084 PMC6067798

[14] Cohen AD , Garfall AL, Stadtmauer EA, Melenhorst JJ, Lacey SF, Lancaster E, . B cell maturation antigen-specific CAR T cells are clinically active in multiple myeloma. J Clin Invest 2019;129:2210–21. 30896447 10.1172/JCI126397 PMC6546468

[15] Xu J , Chen LJ, Yang SS, Sun Y, Wu W, Liu YF, . Exploratory trial of a biepitopic CAR T-targeting B cell maturation antigen in relapsed/refractory multiple myeloma. Proc Natl Acad Sci U S A 2019;116:9543–51. 30988175 10.1073/pnas.1819745116 PMC6510991

[16] Raje N , Berdeja J, Lin Y, Siegel D, Jagannath S, Madduri D, . Anti-BCMA CAR T-cell therapy bb2121 in relapsed or refractory multiple myeloma. N Engl J Med 2019;380:1726–37. 31042825 10.1056/NEJMoa1817226 PMC8202968

[17] Maher J , Brentjens RJ, Gunset G, Riviere I, Sadelain M. Human T-lymphocyte cytotoxicity and proliferation directed by a single chimeric TCRzeta/CD28 receptor. Nat Biotechnol 2002;20:70–5. 11753365 10.1038/nbt0102-70

[18] Imai C , Mihara K, Andreansky M, Nicholson IC, Pui CH, Geiger TL, . Chimeric receptors with 4–1BB signaling capacity provoke potent cytotoxicity against acute lymphoblastic leukemia. Leukemia 2004;18:676–84. 14961035 10.1038/sj.leu.2403302

[19] Mata M , Gottschalk S. Engineering for success: approaches to improve chimeric antigen receptor T cell therapy for solid tumors. Drugs 2019;79:401–15. 30796733 10.1007/s40265-019-01071-7 PMC6613829

[20] Cappell KM , Kochenderfer JN. A comparison of chimeric antigen receptors containing CD28 versus 4–1BB costimulatory domains. Nat Rev Clin Oncol 2021;18:715–27. 34230645 10.1038/s41571-021-00530-z

[21] Wang X , Riviere I. Clinical manufacturing of CAR T cells: foundation of a promising therapy. Mol Ther Oncolytics 2016;3:16015. 27347557 10.1038/mto.2016.15 PMC4909095

[22] Chen N , Li X, Chintala NK, Tano ZE, Adusumilli PS. Driving CARs on the uneven road of antigen heterogeneity in solid tumors. Curr Opin Immunol 2018;51:103–10. 29554494 10.1016/j.coi.2018.03.002 PMC5943172

[23] Globerson Levin A , Riviere I, Eshhar Z, Sadelain M. CAR T cells: Building on the CD19 paradigm. Eur J Immunol 2021;51:2151–63. 34196410 10.1002/eji.202049064 PMC9392049

[24] Irvine DJ , Maus MV, Mooney DJ, Wong WW. The future of engineered immune cell therapies. Science 2022;378:853–8. 36423279 10.1126/science.abq6990 PMC9919886

[25] Majzner RG , Mackall CL. Tumor antigen escape from CAR T-cell therapy. Cancer Discov 2018;8:1219–26. 30135176 10.1158/2159-8290.CD-18-0442

[26] Shah NN , Fry TJ. Mechanisms of resistance to CAR T cell therapy. Nat Rev Clin Oncol 2019;16:372–85. 30837712 10.1038/s41571-019-0184-6 PMC8214555

[27] Hamieh M , Dobrin A, Cabriolu A, van der Stegen SJC, Giavridis T, Mansilla-Soto J, . CAR T cell trogocytosis and cooperative killing regulate tumour antigen escape. Nature 2019;568:112–6. 30918399 10.1038/s41586-019-1054-1 PMC6707377

[28] Majzner RG , Rietberg SP, Sotillo E, Dong R, Vachharajani VT, Labanieh L, . Tuning the antigen density requirement for CAR T-cell activity. Cancer Discov 2020;10:702–23. 32193224 10.1158/2159-8290.CD-19-0945 PMC7939454

[29] Heitzeneder S , Bosse KR, Zhu Z, Zhelev D, Majzner RG, Radosevich MT, . GPC2-CAR T cells tuned for low antigen density mediate potent activity against neuroblastoma without toxicity. Cancer Cell 2022;40:53–69. 34971569 10.1016/j.ccell.2021.12.005 PMC9092726

[30] Priceman SJ , Gerdts EA, Tilakawardane D, Kennewick KT, Murad JP, Park AK, . Co-stimulatory signaling determines tumor antigen sensitivity and persistence of CAR T cells targeting PSCA+ metastatic prostate cancer. Oncoimmunology 2018;7:e1380764. 29308300 10.1080/2162402X.2017.1380764 PMC5749625

[31] Orlando EJ , Han X, Tribouley C, Wood PA, Leary RJ, Riester M, . Genetic mechanisms of target antigen loss in CAR19 therapy of acute lymphoblastic leukemia. Nat Med 2018;24:1504–6. 30275569 10.1038/s41591-018-0146-z

[32] Rabilloud T , Potier D, Pankaew S, Nozais M, Loosveld M, Payet-Bornet D. Single-cell profiling identifies pre-existing CD19-negative subclones in a B-ALL patient with CD19-negative relapse after CAR-T therapy. Nat Commun 2021;12:865. 33558546 10.1038/s41467-021-21168-6 PMC7870924

[33] Li Y , Basar R, Wang G, Liu E, Moyes JS, Li L, . KIR-based inhibitory CARs overcome CAR-NK cell trogocytosis-mediated fratricide and tumor escape. Nat Med 2022;28:2133–44. 36175679 10.1038/s41591-022-02003-x PMC9942695

[34] Olson ML , Mause ERV, Radhakrishnan SV, Brody JD, Rapoport AP, Welm AL, . Low-affinity CAR T cells exhibit reduced trogocytosis, preventing rapid antigen loss, and increasing CAR T cell expansion. Leukemia 2022;36:1943–6. 35490197 10.1038/s41375-022-01585-2 PMC9252916

[35] Salter AI , Ivey RG, Kennedy JJ, Voillet V, Rajan A, Alderman EJ, . Phosphoproteomic analysis of chimeric antigen receptor signaling reveals kinetic and quantitative differences that affect cell function. Sci Signal 2018;11:eaat6753. 30131370 10.1126/scisignal.aat6753 PMC6186424

[36] Mansilla-Soto J , Eyquem J, Haubner S, Hamieh M, Feucht J, Paillon N, . HLA-independent T cell receptors for targeting tumors with low antigen density. Nat Med 2022;28:345–52. 35027758 10.1038/s41591-021-01621-1 PMC9469647

[37] Feucht J , Sun J, Eyquem J, Ho YJ, Zhao Z, Leibold J, . Calibration of CAR activation potential directs alternative T cell fates and therapeutic potency. Nat Med 2019;25:82–8. 30559421 10.1038/s41591-018-0290-5 PMC6532069

[38] Duan Y , Chen J, Meng X, Liu L, Shang K, Wu X, . Balancing activation and co-stimulation of CAR tunes signaling dynamics and enhances therapeutic potency. Mol Ther 2023;31:35–47. 36045585 10.1016/j.ymthe.2022.08.018 PMC9840118

[39] Schoutrop E , Poiret T, El-Serafi I, Zhao Y, He R, Moter A, . Tuned activation of MSLN-CAR T cells induces superior antitumor responses in ovarian cancer models. J Immunother Cancer 2023;11:e005691. 36746513 10.1136/jitc-2022-005691 PMC9906404

[40] Salter AI , Rajan A, Kennedy JJ, Ivey RG, Shelby SA, Leung I, . Comparative analysis of TCR and CAR signaling informs CAR designs with superior antigen sensitivity and in vivo function. Sci Signal 2021;14:eabe2606. 34429382 10.1126/scisignal.abe2606 PMC8613804

[41] Vander Mause ER , Atanackovic D, Lim CS, Luetkens T. Roadmap to affinity-tuned antibodies for enhanced chimeric antigen receptor T cell function and selectivity. Trends Biotechnol 2022;40:875–90. 35078657 10.1016/j.tibtech.2021.12.009

[42] He C , Mansilla-Soto J, Khanra N, Hamieh M, Bustos V, Paquette AJ, . CD19 CAR antigen engagement mechanisms and affinity tuning. Sci Immunol 2023; 8:eadf1426. 10.1126/sciimmunol.adf1426 PMC10228544 36867678

[43] Carnevale J , Shifrut E, Kale N, Nyberg WA, Blaeschke F, Chen YY, . RASA2 ablation in T cells boosts antigen sensitivity and long-term function. Nature 2022;609:174–82. 36002574 10.1038/s41586-022-05126-w PMC9433322

[44] Katsarou A , Sjostrand M, Naik J, Mansilla-Soto J, Kefala D, Kladis G, . Combining a CAR and a chimeric costimulatory receptor enhances T cell sensitivity to low antigen density and promotes persistence. Sci Transl Med 2021;13:eabh1962. 34878825 10.1126/scitranslmed.abh1962 PMC9869449

[45] Gerard A , Cope AP, Kemper C, Alon R, Kochl R. LFA-1 in T cell priming, differentiation, and effector functions. Trends Immunol 2021;42:706–22. 34266767 10.1016/j.it.2021.06.004 PMC10734378

[46] Larson RC , Kann MC, Bailey SR, Haradhvala NJ, Llopis PM, Bouffard AA, . CAR T cell killing requires the IFNgammaR pathway in solid but not liquid tumours. Nature 2022;604:563–70. 35418687 10.1038/s41586-022-04585-5

[47] Chakraborty AK , Weiss A. Insights into the initiation of TCR signaling. Nat Immunol 2014;15:798–807. 25137454 10.1038/ni.2940 PMC5226627

[48] Davis MM , Krogsgaard M, Huppa JB, Sumen C, Purbhoo MA, Irvine DJ, . Dynamics of cell surface molecules during T cell recognition. Annu Rev Biochem 2003;72:717–42. 14527326 10.1146/annurev.biochem.72.121801.161625

[49] Liu Y , Liu G, Wang J, Zheng ZY, Jia L, Rui W, . Chimeric STAR receptors using TCR machinery mediate robust responses against solid tumors. Sci Transl Med 2021;13:eabb5191. 33762437 10.1126/scitranslmed.abb5191

[50] Birtel M , Voss R, Reinhard K, Rengstl B, Ouchan Y, Michel K, . A TCR-like CAR promotes sensitive antigen recognition and controlled T-cell expansion upon mRNA vaccination. Cancer Research Communications 2022;2:827–41. 36923303 10.1158/2767-9764.CRC-21-0154 PMC10010320

[51] Kuwana Y , Asakura Y, Utsunomiya N, Nakanishi M, Arata Y, Itoh S, . Expression of chimeric receptor composed of immunoglobulin-derived V regions and T-cell receptor-derived C regions. Biochem Biophys Res Commun 1987;149:960–8. 3122749 10.1016/0006-291x(87)90502-x

[52] Xu Y , Yang Z, Horan LH, Zhang P, Liu L, Zimdahl B, . A novel antibody-TCR (AbTCR) platform combines Fab-based antigen recognition with gamma/delta-TCR signaling to facilitate T cell cytotoxicity with low cytokine release. Cell Discov 2018;4:62. 30479831 10.1038/s41421-018-0066-6 PMC6242878

[53] Helsen CW , Hammill JA, Lau VWC, Mwawasi KA, Afsahi A, Bezverbnaya K, . The chimeric TAC receptor co-opts the T cell receptor yielding robust anti-tumor activity without toxicity. Nat Commun 2018;9:3049. 30076299 10.1038/s41467-018-05395-y PMC6076291

[54] Baeuerle PA , Ding J, Patel E, Thorausch N, Horton H, Gierut J, . Synthetic TRuC receptors engaging the complete T-cell receptor for potent anti-tumor response. Nat Commun 2019;10:2087. 31064990 10.1038/s41467-019-10097-0 PMC6504948

[55] Burton J , Siller-Farfan JA, Pettmann J, Salzer B, Kutuzov M, van der Merwe PA, . Inefficient exploitation of accessory receptors reduces the sensitivity of chimeric antigen receptors. Proc Natl Acad Sci U S A 2023;120:e2216352120. 36598945 10.1073/pnas.2216352120 PMC9926289

[56] Qu C , Zhang H, Cao H, Tang L, Mo H, Liu F, . Tumor buster - where will the CAR-T cell therapy ‘missile’ go? Mol Cancer 2022;21:201. 36261831 10.1186/s12943-022-01669-8 PMC9580202

[57] Ruella M , Barrett DM, Kenderian SS, Shestova O, Hofmann TJ, Perazzelli J, . Dual CD19 and CD123 targeting prevents antigen-loss relapses after CD19-directed immunotherapies. J Clin Invest 2016;126:3814–26. 27571406 10.1172/JCI87366 PMC5096828

[58] Qin H , Ramakrishna S, Nguyen S, Fountaine TJ, Ponduri A, Stetler-Stevenson M, . Preclinical development of bivalent chimeric antigen receptors targeting both CD19 and CD22. Mol Ther Oncolytics 2018;11:127–37. 30581986 10.1016/j.omto.2018.10.006 PMC6300726

[59] Garfall AL , Cohen AD, Susanibar-Adaniya SP, Hwang WT, Vogl DT, Waxman AJ, . Anti-BCMA/CD19 CAR T cells with early immunomodulatory maintenance for multiple myeloma responding to initial or later-line therapy. Blood Cancer Discov 2023;4:118–33. 36413381 10.1158/2643-3230.BCD-22-0074 PMC9975770

[60] Shalabi H , Qin H, Su A, Yates B, Wolters PL, Steinberg SM, . CD19/22 CAR T-cells in children and young adults with B-ALL: phase I results and development of a novel bicistronic CAR. Blood 2022;140:451–63. 35605184 10.1182/blood.2022015795 PMC9353146

[61] Zah E , Lin MY, Silva-Benedict A, Jensen MC, Chen YY. T cells expressing CD19/CD20 bispecific chimeric antigen receptors prevent antigen escape by malignant B cells. Cancer Immunol Res 2016;4:498–508. 27059623 10.1158/2326-6066.CIR-15-0231 PMC4933590

[62] de Larrea CF , Staehr M, Lopez AV, Ng KY, Chen Y, Godfrey WD, . Defining an optimal dual-targeted CAR T-cell therapy approach simultaneously targeting BCMA and GPRC5D to prevent BCMA escape-driven relapse in multiple myeloma. Blood Cancer Discov 2020;1:146–54. 33089218 10.1158/2643-3230.BCD-20-0020 PMC7575057

[63] Hegde M , Corder A, Chow KK, Mukherjee M, Ashoori A, Kew Y, . Combinational targeting offsets antigen escape and enhances effector functions of adoptively transferred T cells in glioblastoma. Mol Ther 2013;21:2087–101. 23939024 10.1038/mt.2013.185 PMC3831041

[64] Hegde M , Mukherjee M, Grada Z, Pignata A, Landi D, Navai SA, . Tandem CAR T cells targeting HER2 and IL13Ralpha2 mitigate tumor antigen escape. J Clin Invest 2016;126:3036–52. 27427982 10.1172/JCI83416 PMC4966331

[65] Leung I , Templeton ML, Lo Y, Rajan A, Stull SM, Garrison SM, . Compromised antigen binding and signaling interfere with bispecific CD19 and CD79a chimeric antigen receptor function. Blood Adv 2022 Dec 5 [Epub ahead of print]. 10.1182/bloodadvances.2022008559 PMC10275707 36469024

[66] Spiegel JY , Patel S, Muffly L, Hossain NM, Oak J, Baird JH, . CAR T cells with dual targeting of CD19 and CD22 in adult patients with recurrent or refractory B cell malignancies: a phase 1 trial. Nat Med 2021;27:1419–31. 34312556 10.1038/s41591-021-01436-0 PMC8363505

[67] Wang Y , Yang Y, Hong R, Zhao H, Wei G, Wu W, . A retrospective comparison of CD19 single and CD19/CD22 bispecific targeted chimeric antigen receptor T-cell therapy in patients with relapsed/refractory acute lymphoblastic leukemia. Blood Cancer J 2020;10:105. 33077713 10.1038/s41408-020-00371-6 PMC7572410

[68] Larson SM , Walthers CM, Ji B, Ghafouri SN, Naparstek J, Trent J, . CD19/CD20 bispecific chimeric antigen receptor (CAR) in naive/memory T cells for the treatment of relapsed or refractory non-Hodgkin lymphoma. Cancer Discov 2023;13:580–97. 36416874 10.1158/2159-8290.CD-22-0964 PMC9992104

[69] Shah NN , Johnson BD, Schneider D, Zhu F, Szabo A, Keever-Taylor CA, . Bispecific anti-CD20, anti-CD19 CAR T cells for relapsed B cell malignancies: a phase 1 dose escalation and expansion trial. Nat Med 2020;26:1569–75. 33020647 10.1038/s41591-020-1081-3

[70] Cordoba S , Onuoha S, Thomas S, Pignataro DS, Hough R, Ghorashian S, . CAR T cells with dual targeting of CD19 and CD22 in pediatric and young adult patients with relapsed or refractory B cell acute lymphoblastic leukemia: a phase 1 trial. Nat Med 2021;27:1797–805. 34642489 10.1038/s41591-021-01497-1 PMC8516648

[71] Mei H , Li C, Jiang H, Zhao X, Huang Z, Jin D, . A bispecific CAR-T cell therapy targeting BCMA and CD38 in relapsed or refractory multiple myeloma. J Hematol Oncol 2021;14:161. 34627333 10.1186/s13045-021-01170-7 PMC8501733

[72] Bielamowicz K , Fousek K, Byrd TT, Samaha H, Mukherjee M, Aware N, . Trivalent CAR T cells overcome interpatient antigenic variability in glioblastoma. Neuro Oncol 2018;20:506–18. 29016929 10.1093/neuonc/nox182 PMC5909636

[73] Fousek K , Watanabe J, Joseph SK, George A, An X, Byrd TT, . CAR T-cells that target acute B-lineage leukemia irrespective of CD19 expression. Leukemia 2021;35:75–89. 32205861 10.1038/s41375-020-0792-2 PMC7519582

[74] Schneider D , Xiong Y, Wu D, Hu P, Alabanza L, Steimle B, . Trispecific CD19-CD20-CD22-targeting duoCAR-T cells eliminate antigen-heterogeneous B cell tumors in preclinical models. Sci Transl Med 2021;13:eabc6401. 33762438 10.1126/scitranslmed.abc6401

[75] Balakrishnan A , Rajan A, Salter AI, Kosasih PL, Wu Q, Voutsinas J, . Multispecific targeting with synthetic ankyrin repeat motif chimeric antigen receptors. Clin Cancer Res 2019;25:7506–16. 31548346 10.1158/1078-0432.CCR-19-1479 PMC6940018

[76] Wong DP , Roy NK, Zhang K, Anukanth A, Asthana A, Shirkey-Son NJ, . A BAFF ligand-based CAR-T cell targeting three receptors and multiple B cell cancers. Nat Commun 2022;13:217. 35017485 10.1038/s41467-021-27853-w PMC8752722

[77] Hirabayashi K , Du H, Xu Y, Shou P, Zhou X, Fuca G, . Dual targeting CAR-T cells with optimal costimulation and metabolic fitness enhance antitumor activity and prevent escape in solid tumors. Nat Cancer 2021;2:904–18. 34746799 10.1038/s43018-021-00244-2 PMC8570569

[78] Muliaditan T , Halim L, Whilding LM, Draper B, Achkova DY, Kausar F, . Synergistic T cell signaling by 41BB and CD28 is optimally achieved by membrane proximal positioning within parallel chimeric antigen receptors. Cell Rep Med 2021;2:100457. 35028604 10.1016/j.xcrm.2021.100457 PMC8714859

[79] Cho JH , Collins JJ, Wong WW. Universal chimeric antigen receptors for multiplexed and logical control of T cell responses. Cell 2018;173:1426–38. 29706540 10.1016/j.cell.2018.03.038 PMC5984158

[80] Cho JH , Okuma A, Sofjan K, Lee S, Collins JJ, Wong WW. Engineering advanced logic and distributed computing in human CAR immune cells. Nat Commun 2021;12:792. 33542232 10.1038/s41467-021-21078-7 PMC7862674

[81] Choi BD , Yu X, Castano AP, Bouffard AA, Schmidts A, Larson RC, . CAR-T cells secreting BiTEs circumvent antigen escape without detectable toxicity. Nat Biotechnol 2019;37:1049–58. 31332324 10.1038/s41587-019-0192-1

[82] Kloss CC , Condomines M, Cartellieri M, Bachmann M, Sadelain M. Combinatorial antigen recognition with balanced signaling promotes selective tumor eradication by engineered T cells. Nat Biotechnol 2013;31:71–5. 23242161 10.1038/nbt.2459 PMC5505184

[83] Lee S , Khalil AS, Wong WW. Recent progress of gene circuit designs in immune cell therapies. Cell Syst 2022;13:864–73. 36395726 10.1016/j.cels.2022.09.006 PMC9681026

[84] Simon S , Bugos G, Salter AI, Riddell SR. Synthetic receptors for logic gated T cell recognition and function. Curr Opin Immunol 2022;74:9–17. 34571290 10.1016/j.coi.2021.09.003 PMC8901444

[85] Flugel CL , Majzner RG, Krenciute G, Dotti G, Riddell SR, Wagner DL, . Overcoming on-target, off-tumour toxicity of CAR T cell therapy for solid tumours. Nat Rev Clin Oncol 2023;20:49–62. 36418477 10.1038/s41571-022-00704-3 PMC10278599

[86] He X , Feng Z, Ma J, Ling S, Cao Y, Gurung B, . Bispecific and split CAR T cells targeting CD13 and TIM3 eradicate acute myeloid leukemia. Blood 2020;135:713–23. 31951650 10.1182/blood.2019002779 PMC7059518

[87] Lanitis E , Poussin M, Klattenhoff AW, Song D, Sandaltzopoulos R, June CH, . Chimeric antigen receptor T Cells with dissociated signaling domains exhibit focused antitumor activity with reduced potential for toxicity in vivo. Cancer Immunol Res 2013;1:43–53. 24409448 10.1158/2326-6066.CIR-13-0008 PMC3881605

[88] Wilkie S , van Schalkwyk MC, Hobbs S, Davies DM, van der Stegen SJ, Pereira AC, . Dual targeting of ErbB2 and MUC1 in breast cancer using chimeric antigen receptors engineered to provide complementary signaling. J Clin Immunol 2012;32:1059–70. 22526592 10.1007/s10875-012-9689-9

[89] Tousley AM, Rotiroti MC, Labanieh L, Rysavy LW, Kim WJ, Lareau C, et al. Co-opting signalling molecules enables logic-gated control of CAR T cells. Nature 2023;615:507–16. 10.1038/s41586-023-05778-2 PMC10564584 36890224

[90] Fedorov VD , Themeli M, Sadelain M. PD-1- and CTLA-4-based inhibitory chimeric antigen receptors (iCARs) divert off-target immunotherapy responses. Sci Transl Med 2013;5:215ra172. 10.1126/scitranslmed.3006597 PMC4238416 24337479

[91] Richards RM , Zhao F, Freitas KA, Parker KR, Xu P, Fan A, . NOT-Gated CD93 CAR T cells effectively target AML with minimized endothelial cross-reactivity. Blood Cancer Discov 2021;2:648–65. 34778803 10.1158/2643-3230.BCD-20-0208 PMC8580619

[92] Hwang MS , Mog BJ, Douglass J, Pearlman AH, Hsiue EH, Paul S, . Targeting loss of heterozygosity for cancer-specific immunotherapy. Proc Natl Acad Sci U S A 2021;118:e2022410118. 33731480 10.1073/pnas.2022410118 PMC8000272

[93] Sandberg ML , Wang X, Martin AD, Nampe DP, Gabrelow GB, Li CZ, . A carcinoembryonic antigen-specific cell therapy selectively targets tumor cells with HLA loss of heterozygosity in vitro and in vivo. Sci Transl Med 2022;14:eabm0306. 35235342 10.1126/scitranslmed.abm0306

[94] Allen GM , Lim WA. Rethinking cancer targeting strategies in the era of smart cell therapeutics. Nat Rev Cancer 2022;22:693–702. 36175644 10.1038/s41568-022-00505-x

[95] Morsut L , Roybal KT, Xiong X, Gordley RM, Coyle SM, Thomson M, . Engineering customized cell sensing and response behaviors using synthetic notch receptors. Cell 2016;164:780–91. 26830878 10.1016/j.cell.2016.01.012 PMC4752866

[96] Roybal KT , Rupp LJ, Morsut L, Walker WJ, McNally KA, Park JS, . Precision tumor recognition by T cells with combinatorial antigen-sensing circuits. Cell 2016;164:770–9. 26830879 10.1016/j.cell.2016.01.011 PMC4752902

[97] Roybal KT , Williams JZ, Morsut L, Rupp LJ, Kolinko I, Choe JH, . Engineering T cells with customized therapeutic response programs using synthetic notch receptors. Cell 2016;167:419–32. 27693353 10.1016/j.cell.2016.09.011 PMC5072533

[98] Hyrenius-Wittsten A , Su Y, Park M, Garcia JM, Alavi J, Perry N, . SynNotch CAR circuits enhance solid tumor recognition and promote persistent antitumor activity in mouse models. Sci Transl Med 2021;13:eabd8836. 33910981 10.1126/scitranslmed.abd8836 PMC8594452

[99] Choe JH , Watchmaker PB, Simic MS, Gilbert RD, Li AW, Krasnow NA, . SynNotch-CAR T cells overcome challenges of specificity, heterogeneity, and persistence in treating glioblastoma. Sci Transl Med 2021;13:eabe7378. 33910979 10.1126/scitranslmed.abe7378 PMC8362330

[100] Hernandez-Lopez RA , Yu W, Cabral KA, Creasey OA, Lopez Pazmino MDP, Tonai Y, . T cell circuits that sense antigen density with an ultrasensitive threshold. Science 2021;371:1166–71. 33632893 10.1126/science.abc1855 PMC8025675

[101] Zhu I , Liu R, Garcia JM, Hyrenius-Wittsten A, Piraner DI, Alavi J, . Modular design of synthetic receptors for programmed gene regulation in cell therapies. Cell 2022;185:1431–43. 35427499 10.1016/j.cell.2022.03.023 PMC9108009

[102] Williams JZ , Allen GM, Shah D, Sterin IS, Kim KH, Garcia VP, . Precise T cell recognition programs designed by transcriptionally linking multiple receptors. Science 2020;370:1099–104. 33243890 10.1126/science.abc6270 PMC8054651

[103] Srivastava S , Salter AI, Liggitt D, Yechan-Gunja S, Sarvothama M, Cooper K, . Logic-Gated ROR1 chimeric antigen receptor expression rescues T cell-mediated toxicity to normal tissues and enables selective tumor targeting. Cancer Cell 2019;35:489–503. 30889382 10.1016/j.ccell.2019.02.003 PMC6450658

[104] Craigie R , Bushman FD. Host factors in retroviral integration and the selection of integration target sites. Microbiol Spectr 2014;2:10.1128/microbiolspec.MDNA3-0026-2014. PMC4525071 26104434

[105] Ivics Z , Hackett PB, Plasterk RH, Izsvak Z. Molecular reconstruction of Sleeping Beauty, a Tc1-like transposon from fish, and its transposition in human cells. Cell 1997;91:501–10. 9390559 10.1016/s0092-8674(00)80436-5

[106] Fraser MJ , Ciszczon T, Elick T, Bauser C. Precise excision of TTAA-specific lepidopteran transposons piggyBac (IFP2) and tagalong (TFP3) from the baculovirus genome in cell lines from two species of Lepidoptera. Insect Mol Biol 1996;5:141–51. 8673264 10.1111/j.1365-2583.1996.tb00048.x

[107] Singh H , Huls H, Kebriaei P, Cooper LJ. A new approach to gene therapy using Sleeping Beauty to genetically modify clinical-grade T cells to target CD19. Immunol Rev 2014;257:181–90. 24329797 10.1111/imr.12137 PMC4109051

[108] Eyquem J , Mansilla-Soto J, Giavridis T, van der Stegen SJ, Hamieh M, Cunanan KM, . Targeting a CAR to the TRAC locus with CRISPR/Cas9 enhances tumour rejection. Nature 2017;543:113–7. 28225754 10.1038/nature21405 PMC5558614

[109] Nyberg WA , Ark J, To A, Clouden S, Reeder G, Muldoon JJ, . An evolved AAV variant enables efficient genetic engineering of murine T cells. Cell 2023;186:446–60.e19. 36638795 10.1016/j.cell.2022.12.022 PMC10540678

[110] Zhang J , Hu Y, Yang J, Li W, Zhang M, Wang Q, . Non-viral, specifically targeted CAR-T cells achieve high safety and efficacy in B-NHL. Nature 2022;609:369–74. 36045296 10.1038/s41586-022-05140-y PMC9452296

[111] MacLeod DT , Antony J, Martin AJ, Moser RJ, Hekele A, Wetzel KJ, . Integration of a CD19 CAR into the TCR alpha chain locus streamlines production of allogeneic gene-edited CAR T cells. Mol Ther 2017;25:949–61. 28237835 10.1016/j.ymthe.2017.02.005 PMC5383629

[112] Sather BD , Romano Ibarra GS, Sommer K, Curinga G, Hale M, Khan IF, . Efficient modification of CCR5 in primary human hematopoietic cells using a megaTAL nuclease and AAV donor template. Sci Transl Med 2015;7:307ra156. 10.1126/scitranslmed.aac5530 PMC4790757 26424571

[113] Bailey SR , Maus MV. Gene editing for immune cell therapies. Nat Biotechnol 2019;37:1425–34. 31160723 10.1038/s41587-019-0137-8

[114] Ellis GI , Sheppard NC, Riley JL. Genetic engineering of T cells for immunotherapy. Nat Rev Genet 2021;22:427–47. 33603158 10.1038/s41576-021-00329-9 PMC8217325

[115] Torikai H , Reik A, Liu PQ, Zhou Y, Zhang L, Maiti S, . A foundation for universal T-cell based immunotherapy: T cells engineered to express a CD19-specific chimeric-antigen-receptor and eliminate expression of endogenous TCR. Blood 2012;119:5697–705. 22535661 10.1182/blood-2012-01-405365 PMC3382929

[116] Rupp LJ , Schumann K, Roybal KT, Gate RE, Ye CJ, Lim WA, . CRISPR/Cas9-mediated PD-1 disruption enhances anti-tumor efficacy of human chimeric antigen receptor T cells. Sci Rep 2017;7:737. 28389661 10.1038/s41598-017-00462-8 PMC5428439

[117] Qasim W , Zhan H, Samarasinghe S, Adams S, Amrolia P, Stafford S, . Molecular remission of infant B-ALL after infusion of universal TALEN gene-edited CAR T cells. Sci Transl Med 2017;9:eaaj2013. 28123068 10.1126/scitranslmed.aaj2013

[118] Benjamin R , Jain N, Maus MV, Boissel N, Graham C, Jozwik A, . UCART19, a first-in-class allogeneic anti-CD19 chimeric antigen receptor T-cell therapy for adults with relapsed or refractory B-cell acute lymphoblastic leukaemia (CALM): a phase 1, dose-escalation trial. Lancet Haematol 2022;9:e833–e43. 36228643 10.1016/S2352-3026(22)00245-9 PMC11575699

[119] Ottaviano G , Georgiadis C, Gkazi SA, Syed F, Zhan H, Etuk A, . Phase 1 clinical trial of CRISPR-engineered CAR19 universal T cells for treatment of children with refractory B cell leukemia. Sci Transl Med 2022;14:eabq3010. 36288281 10.1126/scitranslmed.abq3010

[120] Stadtmauer EA , Fraietta JA, Davis MM, Cohen AD, Weber KL, Lancaster E, . CRISPR-engineered T cells in patients with refractory cancer. Science 2020;367:eaba7365. 32029687 10.1126/science.aba7365 PMC11249135

[121] Poirot L , Philip B, Schiffer-Mannioui C, Le Clerre D, Chion-Sotinel I, Derniame S, . Multiplex genome-edited T-cell manufacturing platform for “off-the-shelf” adoptive T-cell immunotherapies. Cancer Res 2015;75:3853–64. 26183927 10.1158/0008-5472.CAN-14-3321

[122] Nahmad AD , Reuveni E, Goldschmidt E, Tenne T, Liberman M, Horovitz-Fried M, . Frequent aneuploidy in primary human T cells after CRISPR-Cas9 cleavage. Nat Biotechnol 2022;40:1807–13. 35773341 10.1038/s41587-022-01377-0 PMC7613940

[123] Anzalone AV , Koblan LW, Liu DR. Genome editing with CRISPR-Cas nucleases, base editors, transposases and prime editors. Nat Biotechnol 2020;38:824–44. 32572269 10.1038/s41587-020-0561-9

[124] Yarnall MTN , Ioannidi EI, Schmitt-Ulms C, Krajeski RN, Lim J, Villiger L, . Drag-and-drop genome insertion of large sequences without double-strand DNA cleavage using CRISPR-directed integrases. Nat Biotechnol 2022 Nov 24 [Epub ahead of print]. 10.1038/s41587-022-01527-4 PMC10257351 36424489

[125] Sadelain M , Papapetrou EP, Bushman FD. Safe harbours for the integration of new DNA in the human genome. Nat Rev Cancer 2011;12:51–8. 22129804 10.1038/nrc3179

[126] Odak A , Yuan H, Feucht J, Cantu VA, Mansilla-Soto J, Kogel F, . Novel extragenic genomic safe harbors for precise therapeutic T cell engineering. Blood 2023 Feb 6 [Epub ahead of print]. 10.1182/blood.2022018924 PMC10273162 36745870

[127] Abou-El-Enein M , Elsallab M, Feldman SA, Fesnak AD, Heslop HE, Marks P, . Scalable manufacturing of CAR T cells for cancer immunotherapy. Blood Cancer Discov 2021;2:408–22. 34568831 10.1158/2643-3230.BCD-21-0084 PMC8462122

[128] Ghassemi S , Durgin JS, Nunez-Cruz S, Patel J, Leferovich J, Pinzone M, . Rapid manufacturing of non-activated potent CAR T cells. Nat Biomed Eng 2022;6:118–28. 35190680 10.1038/s41551-021-00842-6 PMC8860360

[129] Park, JH, Palomba ML, Riviere I, Sikder DS, Senechal B, Wang X, et al. A phase I study of CD19-targeted 19(T2)28z1xx CAR T cells in adult patients with relapsed or refractory diffuse large B-cell lymphoma [abstract]. In: Proceedings of the 64th ASH Annual Meeting and Exposition; 2022 Dec 10–13; New Orleans, LA. Washington (DC): American Society of Hematology; 2022. Abstract nr 163.

[130] Svoboda J , Gerson JN, Landsburg DJ, Chong EA, Barta SK, Nasta SW, et al. Interleukin-18 secreting autologous anti-CD19 CAR T-cells (huCART19-IL18) in patients with non-Hodgkin lymphomas relapsed or refractory to prior CAR T-cell therapy [abstract]. In: Proceedings of the 64th ASH Annual Meeting and Exposition; 2022 Dec 10–13; New Orleans, LA. Washington (DC): American Society of Hematology; 2022. Abstract nr 2016.

[131] Yang J , He J, Zhang X, Li J, Wang Z, Zhang Y, . Next-day manufacture of a novel anti-CD19 CAR-T therapy for B-cell acute lymphoblastic leukemia: first-in-human clinical study. Blood Cancer J 2022;12:104. 35798714 10.1038/s41408-022-00694-6 PMC9262977

[132] Yang J, Jiang P, Zhang X, Li J, Wu Y, Xu L, et al. Successful 24-hours manufacture of anti-CD19/CD22 dual chimeric antigen receptor (CAR) T cell therapy for B-cell acute lymphoblastic leukemia (B-ALL) [abstract]. In: Proceedings of the 62nd ASH Annual Meeting and Exposition; 2020 Dec 5–8; San Diego, CA. Washington (DC): American Society of Hematology; 2020. Abstract nr 159.

[133] Biasco L , Izotova N, Rivat C, Ghorashian S, Richardson R, Guvenel A, . Clonal expansion of T memory stem cells determines early anti-leukemic responses and long-term CAR T cell persistence in patients. Nat Cancer 2021;2:629–42. 34345830 10.1038/s43018-021-00207-7 PMC7611448

[134] Arcangeli S , Bove C, Mezzanotte C, Camisa B, Falcone L, Manfredi F, . CAR T cell manufacturing from naive/stem memory T lymphocytes enhances antitumor responses while curtailing cytokine release syndrome. J Clin Invest 2022;132:e150807. 35503659 10.1172/JCI150807 PMC9197529

[135] Pilipow K , Scamardella E, Lugli E. Generating stem-like memory T cells with antioxidants for adoptive cell transfer immunotherapy of cancer. Methods Enzymol 2020;631:137–58. 31948545 10.1016/bs.mie.2019.08.016

[136] Dreyzin A , Panch SR, Shalabi H, Yates B, Highfill SL, Jin P, . Cryopreserved anti-CD22 and bispecific anti-CD19/22 CAR T cells are as effective as freshly infused cells. Mol Ther Methods Clin Dev 2023;28:51–61. 36620075 10.1016/j.omtm.2022.12.004 PMC9798176

[137] van der Stegen SJC , Lindenbergh PL, Petrovic RM, Xie H, Diop MP, Alexeeva V, . Generation of T-cell-receptor-negative CD8alphabeta-positive CAR T cells from T-cell-derived induced pluripotent stem cells. Nat Biomed Eng 2022;6:1284–97. 35941192 10.1038/s41551-022-00915-0 PMC9669107

[138] Stephan MT . Empowering patients from within: Emerging nanomedicines for *in vivo* immune cell reprogramming. Semin Immunol 2021;56:101537. 34844835 10.1016/j.smim.2021.101537 PMC8792224

[139] Michels A , Ho N, Buchholz CJ. Precision medicine: *In vivo* CAR therapy as a showcase for receptor-targeted vector platforms. Mol Ther 2022;30:2401–15. 35598048 10.1016/j.ymthe.2022.05.018 PMC9263322

[140] Rurik JG , Tombacz I, Yadegari A, Mendez Fernandez PO, Shewale SV, Li L, . CAR T cells produced *in vivo* to treat cardiac injury. Science 2022;375:91–6. 34990237 10.1126/science.abm0594 PMC9983611

